# Early dorsomedial tissue interactions regulate gyrification of distal neocortex

**DOI:** 10.1038/s41467-019-12913-z

**Published:** 2019-11-15

**Authors:** Victor V. Chizhikov, Igor Y. Iskusnykh, Ekaterina Y. Steshina, Nikolai Fattakhov, Anne G. Lindgren, Ashwin S. Shetty, Achira Roy, Shubha Tole, Kathleen J. Millen

**Affiliations:** 1 Department of Anatomy and Neurobiology, University of Tennessee Health Science Center, Memphis, TN 38163 USA; 2 Department of Human Genetics, University of Chicago, Chicago, IL 60637 USA; 3 Department of Biological Sciences, Tata Institute of Fundamental Research, Mumbai, India; 4 Center for Integrative Brain Research, Seattle Children’s Research Institute, Seattle, WA 98101 USA; 5 Department of Pediatrics, University of Washington, Seattle, WA 98101 USA

**Keywords:** Developmental neurogenesis, Cell type diversity

## Abstract

The extent of neocortical gyrification is an important determinant of a species’ cognitive abilities, yet the mechanisms regulating cortical gyrification are poorly understood. We uncover long-range regulation of this process originating at the telencephalic dorsal midline, where levels of secreted Bmps are maintained by factors in both the neuroepithelium and the overlying mesenchyme. In the mouse, the combined loss of transcription factors Lmx1a and Lmx1b, selectively expressed in the midline neuroepithelium and the mesenchyme respectively, causes dorsal midline Bmp signaling to drop at early neural tube stages. This alters the spatial and temporal Wnt signaling profile of the dorsal midline cortical hem, which in turn causes gyrification of the distal neocortex. Our study uncovers early mesenchymal-neuroepithelial interactions that have long-range effects on neocortical gyrification and shows that lissencephaly in mice is actively maintained via redundant genetic regulation of dorsal midline development and signaling.

## Abstract (web summary)

The contribution of long-range signaling to cortical gyrification remains poorly understood. In this study, authors demonstrate that the combined genetic loss of transcription factors Lmx1a and Lmx1b, expressed in the telencephalic dorsal midline neuroepithelium and head mesenchyme, respectively, induces gyrification in the mouse neocortex

## Introduction

Neocortical gyrification, defined as folding of the outer pial surface and underlying neuronal layers of the brain but a smooth ventricular surface, results in an uneven regional thickness of the neocortex and is associated with enhanced species’ cognitive abilities^1–4^. Neocortical gyrification is a variable trait across mammalian species ranging from complete absence of gyrification in lissencephalic mammals, such as the mouse, to highly elaborate gyrification in many primates, such as humans. It is believed that the most recent common mammalian ancestor had a gyrencephalic (folded) cortex and that several transitions between gyrencephaly and lissencephaly (smooth cortex) happened during mammalian evolution as adaptations to each species’ environment and lifestyle^5–7^.

Recent studies have revealed a coordinated sequence of neurogenic events in the developing neocortex of naturally gyrencephalic mammals, which begins with an early accumulation of apical cortical progenitors (APs) in the neocortical ventricular zone (VZ), followed by expansion of basal progenitors (BPs), including intermediate progenitors (IPs) and basal radial glia (bRG). BPs arise from APs and expand in the emerging gyri. It is believed that extensive generation of BPs, especially bRG, at the peak of neurogenesis and the subsequent production of neurons from these progenitors is a major force driving cortical gyrification in higher mammals^3,8–12^. A number of genes that promote gyrification acting locally in cortical progenitors have been identified^4,13–21^. During development, however, many cortical features are regulated by both locally acting mechanisms and long-range signaling. The contribution of signaling molecules, in particular long-range signaling molecules, to cortical gyrification remains poorly understood. Shh and Fgf signaling are known to promote cortical gyrification^16,22–24^. In humans, Shh is expressed and acts locally in cortical progenitors, while the sources of gyrification-related Fgf signaling remain unknown^16,22^.

Although there have been extensive efforts devoted to identify the mechanisms that promote cortical folding in gyrencephalic mammals, the endogenous mechanisms that maintain lissencephaly (suppress gyrification) in naturally lissencephalic mammals have received little attention and remain virtually unknown. One possibility is that lissencephalic species simply lack genes or regulatory elements necessary to drive cortical gyrification^13–15,18,19^. Alternatively, genes in the genome of lissencephalic species actively maintain lissencephaly, suppressing gyrification. To date, several mouse models with a gyrencephalic neocortex have been described. The vast majority of these, however, have been generated via local or transient manipulation of gene function and, therefore, alone are insufficient to dissect the endogenous mechanisms that maintain lissencephaly in the mouse brain. The only exception is the *Flrt1−/−;Flrt3−/−* mouse model^25^. Dual loss of the adhesion genes *Flrt1* and *Flrt3* affects migration of differentiating neurons and results in folding of the mouse neocortex. In this mouse model, however, cortical folding develops without the predominant expansion of BPs observed in higher mammals^25^.

While studying the role of transcription factors Lmx1a and Lmx1b in the cerebellum^26^, we observed that *Lmx1a/b* double, but not single, mutants had unexpected neocortical gyrification. We found that neither *Lmx1a* nor *Lmx1b* were expressed in the neocortex. In contrast to more posterior central nervous system^26^, in the telencephalon, these two genes were not even coexpressed: *Lmx1a* was expressed in the telencephalic dorsal midline neuroepithelium (DMe), while *Lmx1b* was expressed in head mesenchyme. Cortical gyrification in *Lmx1a−/−;b−/−* mutants was associated with expansion of neocortical BPs and resulted from the disruption of spatial and temporal dynamics of Bmp and Wnt signaling cascades originating at the distantly located dorsomedial telencephalon. Our study identifies an unexpected role of dorsal midline signaling in the long-range regulation of cortical gyrification and shows that mesenchymal–neuroepithelial interactions are necessary to maintain lissencephaly in mice.

## Results

### Cortical gyrification in *Lmx1a−/−;b−/−* mice

**Fig. 1:**
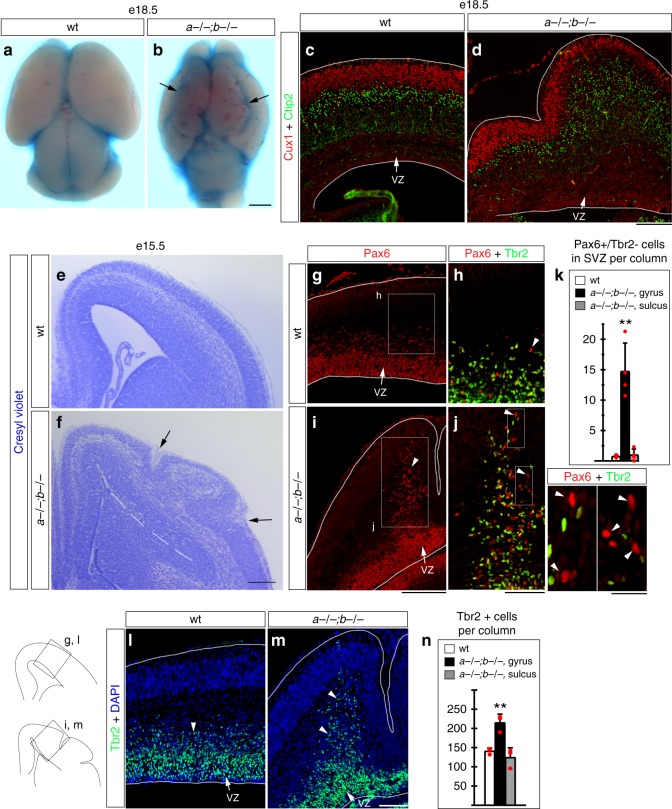
Gyrification with BPs expansion in the *Lmx1a−/−;b−/−* mouse cortex. Dorsal view of whole mount telencephalon with fast green dye applied on the telencephalic surface (**a**, **b**) and coronal cresyl violet or antibody-stained sections (**c**–**j**, **l**, **m**) at indicated stages. **a**, **b** Cortical surface of e18.5 *Lmx1a−/−;b−/−* mutants, but not wild-type littermates, showed groves (arrows) that extended along the anterior-posterior axis in each cortical hemisphere. **c**, **d** Cux1/Ctip2-immunostained sections showing that despite gyrification of the outer cortical surface (faces up), cortical layering was not grossly disrupted in *Lmx1a−/−;b−/−* mutants. Corresponding ventricular surface (faces down) was not folded. VZ - ventricular zone. **e**, **f** Arrows point to sulci that develop on the outer cortical surface of *Lmx1a−/−;b−/−* embryos at e15.5 (**f**). Dashed line demarcates cortical ventricular surface and lateral ganglionic eminence. **g**–**k** Numerous basally located Pax6+ cells were present in the emerging gyri (**i**, arrowhead) but not sulci in e15.5 *Lmx1a−/−;b−/−* mutants (**i**). The number of Pax6+/Tbr2− cells located basal to the dense band of Tbr2 + IPs (arrowheads in **h**, **j**, **j insets**) was dramatically increased in cortical gyri but not sulci relative to wild-type littermates (**p < 0.01, *n* = 4 embryos per genotype). **l–n** In e15.5 *Lmx1a−/−;b−/−* mutants, Tbr2 + IPs (arrowheads) were increased in the number in cortical gyri but not sulci relative to wild-type littermates (**p < 0.01, *n* = 3 embryos per genotype). All data are mean ± s.d., all *p* values are from two-tailed *t*-test after Bonferroni correction for multiple comparisons. Source data for panels **k** and **n** are provided as Source Data File. Scale bars: 1 mm (**a**, **b**); 200 μm (**c**–**g**, **i**); 100 μm (**h**, **j**, **l**, **m**); 25 μm (insets in panel **j**)

Transcription factors Lmx1a and Lmx1b redundantly regulate development of several cellular populations during embryonic development, such as the hindbrain roof plate and midbrain dopaminergic neurons^26,27^. While studying the role of *Lmx1* genes in cerebellar development^26^, we found that *Lmx1a−/−;b−/−* mutants had unexpected and striking gyrification of the neocortex (Fig. 1a–f, Supplementary Fig. 1a–d). All 12 *Lmx1a−/−;b−/−* mutants that we dissected at late embryonic stages had macroscopic cortical folds apparent in both left and right hemispheres (Fig. 1b) yet the cortex remained lissencephalic in littermates with loss of either single gene (*n* = 20–25 mutants for each genotype) (Supplementary Fig. 2). Analysis of postnatal stages was precluded by neonatal death caused by a requirement for *Lmx1b* during mid/hindbrain development^26^. To compare location of cortical gyri in different e18.5 embryos, we divided the neocortical ventricular surface into 10 equally sized segments and measured cortical thickness in the middle of each of these segments (Supplementary Fig. 1e). Average cortical thickness was similar throughout wild-type cortex yet was uneven between different regions of *Lmx1a−/−;b−/−* neocortex, illustrating non-random locations of cortical gyri (Supplementary Fig. 1g, h). In both hemispheres, the most prominent was a dorsal gyrus located in cortical segments 2 (and also in segment 3 in right hemisphere), while the neuroepithelium in segment 4 (and also in segments 5 and 6 in right hemisphere) was thinner. More ventral gyri were less prominent (Supplementary Fig. 1b–d, h). Consistent with cortical gyrification, neocortical area (measured between the dorsal cortical bend and lateral ganglionic eminence, LGE) was increased in *Lmx1a−/−;b−/−* mutants compared to wild-type littermates (Supplementary Fig. 1f).

Similar to wild-type littermates^28^, in *Lmx1a−/−;b−/−* mutants, Cux1+ (upper layer) neurons occupied more superficial positions relative to Ctip2+ (lower layer) neurons, revealing largely preserved cortical lamination (Fig. 1c, d). Thus, *Lmx1a* and *Lmx1b* function redundantly to maintain lissencephaly in the mouse, and simultaneous loss of these genes is sufficient to induce gyrification of the mouse cortex.

### Aberrant expansion of progenitors in *Lmx1a−/−;b−/−* cortex

To determine whether cortical gyrification in *Lmx1a−/−;b−/−* mutants was associated with expansion of cortical BPs, we analyzed embryos at e15.5, during mid-neurogenesis, when *Lmx1a−/−;b−/−* double mutant cortex first displays evidence of gyrification (cortical plate folding relative to ventricular surface) (Fig. 1e, f). In the emerging cortical gyri, but not sulci, of e15.5 *Lmx1a−/−;b−/−* mutants, more Pax6+/Tbr2− and Tbr2+ cells were observed in basal locations relative to wild-type controls (Fig. 1g–n), consistent with focal expansion of bRG and IPs^16,29,30^.

Since proliferation of BPs and the subsequent production of neurons from these progenitors are major mechanisms of cortical gyrification in higher mammals^3,8–12^, we studied BPs in the emerging cortical gyri of *Lmx1a−/−;b−/−* mutants in more detail. Co-labeling with a progenitor marker Ki67 confirmed that basal Pax6+/Tbr2− and Tbr2+ cells in *Lmx1a−/−;b−/−* mutants were indeed progenitors (Supplementary Fig. 3a, b). A short (90 min) BrdU pulse was used to label proliferating cells in the S-phase of the cell cycle. This analysis revealed numerous basally located BrdU+ cells in the emerging gyri of *Lmx1a−/−;b−/−* embryos (Supplementary Fig. 3c, g, arrowheads). The fraction of basally located BrdU+ cells was significantly higher in the emerging gyri of *Lmx1a/b* double mutants compared to wild-type controls (Supplementary Fig. 3c, g, k) arguing that similar to naturally gyrencephalic mammals^3,9,11^, in *Lmx1a−/−;b−/−* cortical gyri, proliferation was shifted to basal positions. Triple BrdU/Pax6/Tbr2 labeling revealed that in *Lmx1a−/−;b−/−* mutants, many BPs incorporated BrdU and, thus, were proliferating (Supplementary Fig. 3c–e, g–i). There was a significant increase in the numbers of both S-phase BrdU+/Pax6+/Tbr2− bRG and BrdU+/Tbr2+ IPs, but not apical BrdU+/Pax6+ cells in cortical gyri of *Lmx1a−/−;b−/−* mutants (Supplementary Fig. 3c–f, g–j, l–n). Since Pax6+/Tbr2− bRG cells were very rare in wild-type embryos, we were unable to compare the fraction of BrdU+ (S-phase) bRG cells between wild-type and *Lmx1a−/−;b−/−* embryos. However, we found that a similar fraction (~20%) of Tbr2+ IPs were BrdU+ (S-phase) in both wild-type and *Lmx1a−/−;b−/−* cortex (Supplementary Fig. 3o), suggesting a similar proliferation rate^31^ of IPs in wild-type and *Lmx1a−/−;b−/−* mutants.

A longer (20 h) BrdU pulse revealed an increased fraction of progenitors exiting the cell cycle in nascent gyri of *Lmx1a−/−;b−/−* embryos (Supplementary Fig. 3p–r), consistent with increased neurogenesis^23^. Analysis of *Lmx1a−/−;b−/−* mutants at e15.5, the time of gyri development, revealed a similar density of apoptotic (activated Caspase 3+) cells in cortical areas producing gyri and sulci (Supplementary Fig. 4), indicating that localized increase in apoptosis is not a major mechanism of cortical gyrification in *Lmx1a−/−;/b−/−* mutants. Thus, cortical gyrification in *Lmx1a−/−;b−/−* mutants was associated with expansion of cortical BPs and increased neurogenesis.

**Fig. 2:**
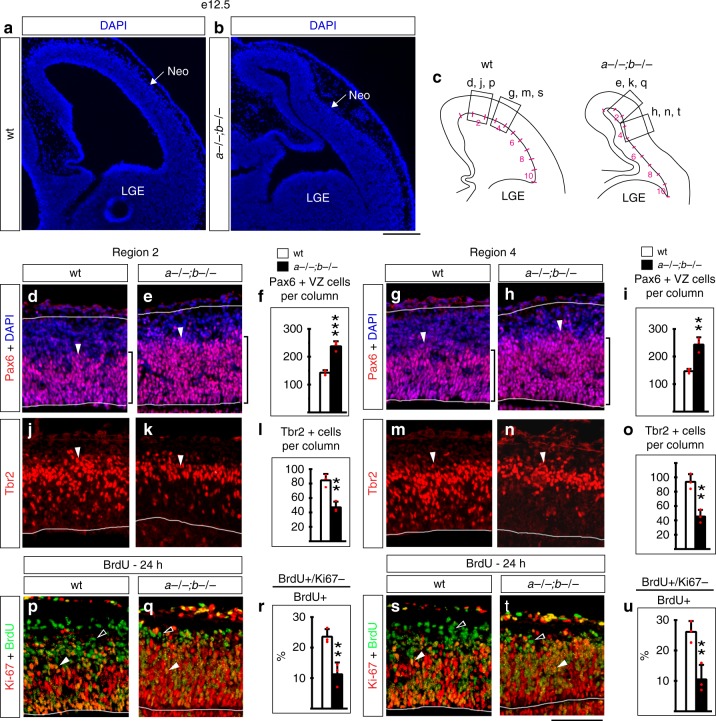
Accumulation of APs precedes gyrification of the *Lmx1a−/−;b−/−* cortex. Coronal sections at e12.5. **a**, **b** No evidence of cortical gyrification (outgrowths on outer cortical surface without bending of the corresponding ventricular surface) was detected in *Lmx1a−/−;b−/−* mutants at e12.5. Neo-neocortical primordium. LGE lateral ganglionic eminence. **c** Ventricular surface in wild-type and *Lmx1a−/−;b−/−* neocortical primordium (between the dorsal cortical bend and LGE) was subdivided into 10 equally sized regions (segments 1–10) and cells were quantified in segment 2 (**d**–**f**, **j**–**l**, **p**–**r**) and segment 4 (**g**–**i**, **m**–**o**, **s**–**u**). **d**–**i** Pax6+ APs (**d**, **e**, **g**, **h,** arrowheads) were increased in the number (**f, i**) (***p < 0.001 and **p < 0.01) and Pax6+VZ zone (bracket) was thicker in *Lmx1a−/−b−/−* mutants in both segments of the neocortical primordium. **j**–**o** Tbr2+ IPs (**j**, **k**, **m**, **n**, arrowheads) were reduced in the number (**l**, **o**) (**p < 0.01) in *Lmx1a−/−;b−/−* mutants in both segments of the neocortical primordium. **p**–**u** Mice were injected with BrdU 24 h prior to collecting embryos. **p**, **q**, **s**, **t** Arrowheads point to cells that re-entered the cell cycle (BrdU+/Ki67+ cells), open arrowheads—to cell that exited the cell cycle (BrdU + /Ki67− cells). A lower fraction of cells exited the cell cycle (the number of BrdU+/Ki67− cells divided by the number of BrdU+ cells) (**p < 0.01) in *Lmx1a−/−;b−/−* mutants in both segments of the neocortical primordium. All data are mean ± s.d., all *p* values are from two-tailed *t*-test, *n* = 3 embryos per genotype. Source data for panels **f**, **i**, **l**, **o**, **r**, **u** are provided as Source Data File. Scale bars: 200 μm (**a**, **b**); 100 μm (all other panels)

Next, we analyzed e12.5 embryos, at the beginning of cortical neurogenesis when *Lmx1a−/−;b−/−* mutants were not yet gyrified (Fig. 2a, b). We studied progenitor populations in two cortical regions—cortical segment 2 (the most consistent location of a future gyrus) and cortical segment 4 (a region with the lowest cortical thickness at e18.5) (Fig. 2c, Supplementary Fig. 1h). At this early stage, compared to controls, both cortical regions of the double mutants had increased numbers of Pax6+ APs, increased radial thickness of the VZ (Fig. 2d–i), reduced numbers of Tbr2+ IPs (Fig. 2j–o), and reduced cell-cycle exit (Fig. 2p–u). Thus, in *Lmx1a/b* double mutants, global overproduction of APs precedes local accumulation of BPs at later stages.

### *Lmx1a/b* are not coexpressed in the developing telencephalon

**Fig. 3:**
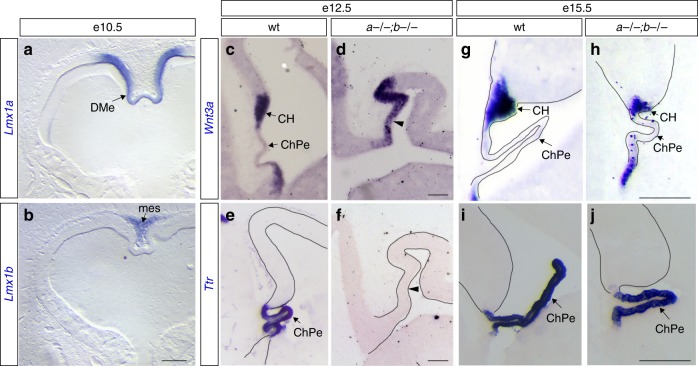
Dual loss of complementary expressed *Lmx1a/b* causes transient DMe mispatterning. **a**, **b** In situ stained wild-type coronal telencephalic sections at e10.5 showing specific expression of *Lmx1a* in the DMe and *Lmx1b*—in medial mesenchyme (mes.). **c**–**f** At e12.5, wild-type CH expressed *Wnt3a* (**c**), while ChPe—*Ttr* (**e**). In *Lmx1a−/−;b−/−* mutants, *Wnt3a* expression domain was enlarged, expanding into the prospective ChPe territory (**d**, arrowhead), while *Ttr* was not expressed (**f**, arrowhead). **g**–**j** DMe patterning was restored in e15.5 *Lmx1a−/−;b−/−* mutants, with *Ttr* expression in the ChPe and *Wnt3a*—in the CH. *Wnt3a*+ CH domain, however, was abnormally small (**g**, **h**). Scale bars: 200 μm (**a**, **b**, **g**, **h**); 150 μm (**c**–**f**); 250 μm (**i**, **j**)

We expected the *Lmx1a* and *Lmx1b* to be coexpressed in the developing telencephalon, based on their co-expression in other cellular populations redundantly regulated by these genes^26,27^. Our extensive in situ hybridization, immunohistochemical and fate-mapping analyses revealed, however, that these genes are never coexpressed in the developing telencephalon. Further, neither is even expressed in neocortical neuroepithelium. *Lmx1a* expression was always restricted to the telencephalic DMe, beginning at the tips of the neural folds at e8.5 and through differentiation of the DMe into the medial choroid plexus epithelium (ChPe) and the cortical hem (CH). *Lmx1b* expression was restricted to the head mesenchyme, including the mesenchyme located adjacent to the DMe (Fig. 3a, b, Supplementary Figs. 5 and 6). Loss of one *Lmx1* gene did not alter expression pattern of the remaining *Lmx1* gene (Supplementary Fig. 7). When considered together with the *Lmx1a−/−;b−/−* cortical phenotype, the *Lmx1a* and *Lmx1b* expression pattern suggests that redundant *Lmx1a/b*-dependent mesenchymal and DMe signaling pathways regulate gyrification of distal neocortex.

### Aberrant DMe and Wnt signaling in *Lmx1a−/−;b−/−* mutants

By e12.5, the wild-type telencephalic DMe differentiates into the medial *Ttr*+ ChPe and adjacent CH that expresses secreted Wnt3a^32–34^ (Fig. 3c, e). In *Lmx1a−/−;b−/−* mutants at this stage, however, *Ttr* expression was absent in a thickened DMe while *Wnt3a* expression expanded across the presumptive ChPe, resulting in an enlarged *Wnt3a* expression domain at the dorsal midline compared to controls (Fig. 3c-f). This phenotype was not observed in either single-gene mutant (Supplementary Fig. 8). In double mutants, the thickened medial neuroepithelium showed increased proliferation in the presumptive ChPe as early as at e10.5 (Supplementary Fig. 9a–g). By e15.5, there was partial amelioration of *Lmx1a−/−;b−/−* DMe deficits: *Ttr* ChPe expression was present and *Wnt3a* expression was restricted to the CH (Fig. 3g–j), although ChPe proliferation was still elevated compared to controls (Supplementary Fig. 9h, i). The double mutant CH also displayed increased apoptosis (Supplementary Fig. 9j–l), resulting in a small *Wnt3a*+ CH at e15.5 (Fig. 3g, h).

Early expansion of *Wnt3a*+ CH in e12.5 *Lmx1a−/−;b−/−* mutants was associated with elevated nuclear β-catenin, a definitive readout of canonical Wnt signaling^35^ (Supplementary Fig. 10) and elevated expression of downstream Wnt-β-catenin target genes *Lef1* and *Axin2*^36^ in the neocortical primordium (Supplementary Fig. 11). However, at e15.5, consistent with the dramatic reduction of size of the double-mutant *Wnt3a*+ CH, nuclear β-catenin was undetectable in the *Lmx1a−/−;b−/−* neocortex, and *Lef1* and *Axin2* levels were significantly reduced relative to comparably staged controls (Supplementary Fig. 12). These data define two distinct phases of canonical Wnt signaling and reception in the *Lmx1a−/−;b−/−* embryonic telencephalon: an increased early phase and a decreased late phase.

In addition to producing Wnt signaling molecules, the early DMe (both the CH and ChPe) gives rise to Cajal-Retzius (CR) cells, a transient neuronal population that migrates along the outer surface of cortical neuroepithelium and regulates cortical development^37,38^. In *Lmx1a−/−;b−/−* mutants, similar to wild-type embryos, CR cells uniformly covered the neocortical primordium, suggesting that cortical gyrification in these mutants does not directly arise from CR cells migratory defects (Supplementary Fig. 13). Thus, to define the mechanism of *Lmx1a−/−;b−/−* cortical gyrification, we focused on misregulated DMe-derived Wnt signaling.

### *Lmx1a−/−;b−/−* cortical gyrification depends on Wnt signals

Wnt/β-catenin signaling promotes expansion of cortical APs and suppresses the generation of BPs from APs^29,31,39^. Early constitutive ablation of β-catenin in the developing telencephalon increased the numbers of both bRG and IPs^29^, the phenotype we also observed in *Lmx1a−/−;b−/−* mutants. However, in contrast to *Lmx1a−/−;b−/−* embryos, β-catenin-ablated mice did not show cortical gyrification, and IP expansion in these mice was observed only at the beginning (e12.5) but not in the middle (e14.5) of neurogenesis, likely due to precocious depletion of the AP pool^29^.

**Fig. 4:**
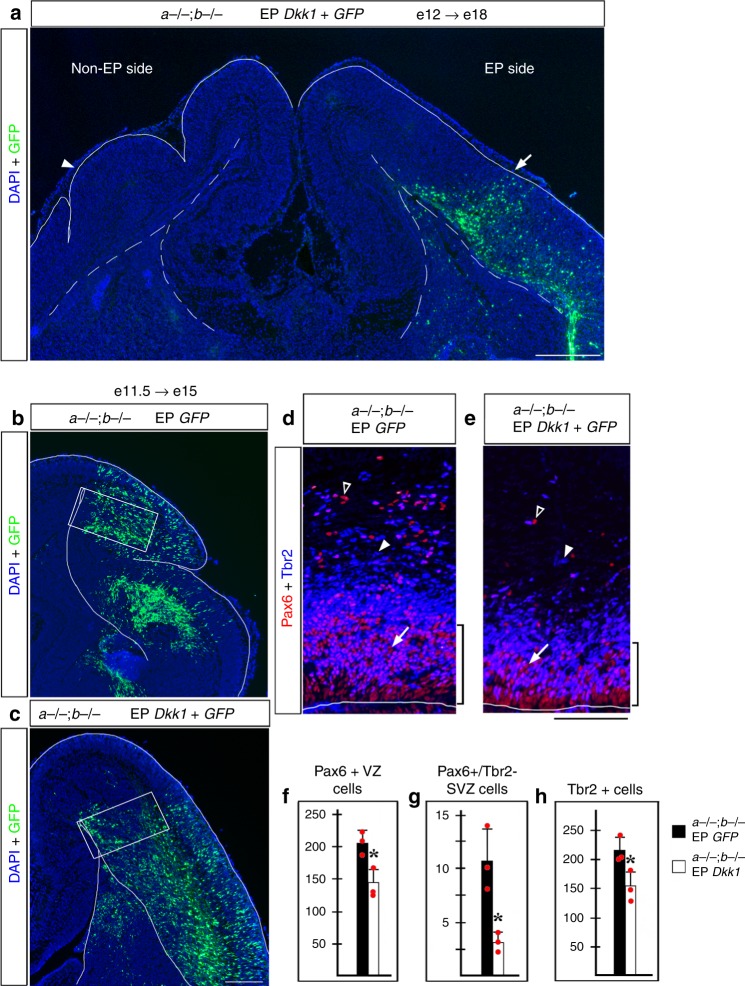
Electroporation of *Dkk1* suppresses *Lmx1a−/−;b−/−* cortical gyrification. **a** An *Lmx1a−/−;b−/−* embryo in utero electroporated with *Dkk1+* *GFP* at e12 and analyzed at e18. Early electroporation of *Dkk1* into neocortical primordium restored lissencephaly (arrow), while the non-electroporated side remained gyrencephalic (arrowhead). **b**–**h** *Lmx1a−/−;b−/−* mutants electroporated with *Dkk1*+ *GFP* or *GFP* alone at e11.5 and analyzed at e15. Panels **d**, **e** are from sections adjacent to those shown in **b**, **c** and correspond to regions boxed in **b**, **c**. **d**–**h** In *Dkk1*-electroporated double mutants, the number of Pax6+ APs (arrows), Pax6+ /Tbr2− bRGs (open arrowheads) and Tbr2+ IPs (arrowheads) was reduced compared to *GFP*-electroporated double mutants (*p < 0.05, two-tailed *t*-test, *n* = 3 *Dkk1*-electroporated and *n* = 3 *GFP*-electroporated *Lmx1a−/−;b−/−* mutants). Bracket (**d**, **e**) indicates thickness of the Pax6+ VZ, which was also reduced in *Dkk1*-electroporated *Lmx1a−/−;b−/−*mutants. All data are mean ± s.d. Source data for panels **f**–**h** are provided as Source Data File. Scale bars: 400 μm (**a**); 200 μm (**b**, **c**); 100 μm (**d**, **e**)

A key difference between β-catenin-ablated mice and our *Lmx1a−/−;b−/−* mutant embryos is that in contrast to β-catenin-ablated mice, in *Lmx1a−/−;b−/−* mutants, a dynamic decrease in Wnt-β-catenin signaling followed its transient overactivation and the early overexpansion of APs. To balance early high levels of Wnt signaling in *Lmx1a−/−;b−/−* neocortex, we electroporated a plasmid encoding Dkk1, a secreted Wnt inhibitor^39^ into the e12 *Lmx1a−/−;b−/−* neocortical primordium. This was sufficient to rescue the gyrification phenotype and restore a lissencephalic cortex on the electroporated side, when assessed at e18 (Fig. 4a). *Dkk1*-electroporated *Lmx1a−/−;b−/−* hemispheres had significantly lower numbers of APs, bRG, and IPs compared to *Lmx1a−/−;b−/−* cortices electroporated with GFP alone (Fig. 4b–h). Thus, we conclude that aberrant dynamics of Wnt signaling is a major contributor to neocortical gyrification in *Lmx1a−/−;b−/−* mice.

### *Lmx1a/b* regulate the DMe Wnt source via Bmp signaling

**Fig. 5:**
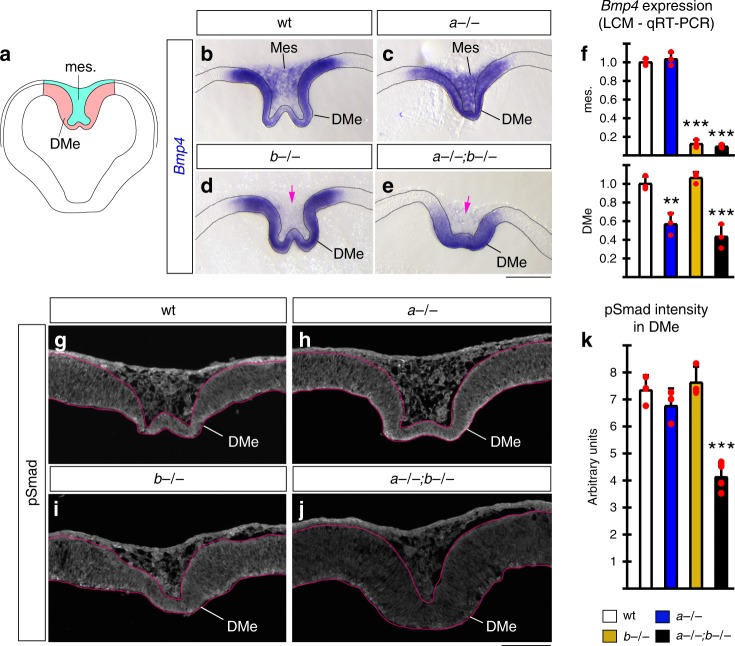
*Lmx1a* and *Lmx1b* regulate Bmp signaling by controlling expression of *Bmp4*. **a** Schematic of e10.5 coronal telencephalic section showing DMe (red) and medial mesenchyme (green). **b**–**e** High magnification images of dorsal midline region of e10.5 telencephalic coronal sections stained with *Bmp4* in situ probe. (Low magnification images of the same sections are shown in Supplementary Fig. 14.) **b** In wild-type telencephalon, *Bmp4* was expressed in the DMe and medial mesenchyme adjacent to DMe (mes.). **c**–**e** Loss of *Lmx1b* abolished *Bmp4* expression in medial mesenchyme (pink arrows in **d**, **e**) but not DMe. Based on in situ, loss of *Lmx1a* did not obviously reduce *Bmp4* expression. **f** qRT-PCR analysis of DMe (red region in panel **a**) and medial mesenchyme (green region in panel **a**) isolated by laser capture microdissection (LCM). Based on qRT-PCR, loss of *Lmx1b* reduced expression of *Bmp4* in medial mesenchyme (mes.) (***p < 0.001 for *Lmx1b*−/− versus wild type and *Lmx1a*−/−;*b−/−* versus wild-type comparisons, one-way ANOVA with Tukey’s post hoc test, *n* = 3 embryos per genotype). Loss of *Lmx1a* reduced expression of *Bmp4* in the DMe (**p < 0.01 for *Lmx1a*−/− versus wild type and ***p = 0.001 for *Lmx1a*−/−;*b−/−* versus wild-type comparisons, one-way ANOVA with Tukey’s post hoc test, *n* = 3 embryos per genotype). **g**–**j** pSmad immunohistochemistry on e10.5 coronal sections. Only simultaneous, but not individual loss of *Lmx1a* and *1b* significantly reduced pSmad intensity in the DMe (***p ≤ 0.001 for *Lmx1a*−/−;*b−/−* versus wild type, *Lmx1a*−/−;*b−/−* versus *Lmx1a*−/−, and *Lmx1a*−/−;*b−/−* versus *Lmx1b*−/− comparisons, one-way ANOVA with Tukey’s post hoc test, *n* = 3 wild-type embryos, *n* = 3 *Lmx1a*−/− embryos, *n* = 3 *Lmx1b*−/− embryos, and *n* = 4 *Lmx1a*−/−;*b−/−* embryos). All data are mean ± s.d. Source data for panels **f**, **k** are provided as Source Data File. Scale bars: 200 μm (**b**–**e**); 100 μm (**g**–**j**)

Next, we focused our attention on the early development of the DMe Wnt source. We hypothesized that *Lmx1b* regulates expression of secreted factors that diffuse from the medial head mesenchyme and cooperate with a *Lmx1a*-dependent pathway in the DMe to regulate the temporal and spatial patterning of the ChPe/CH. Using a candidate gene approach, in situ hybridization revealed *Bmp4* to be expressed in both the *Lmx1b* (mesenchymal) and *Lmx1a* (DMe) territory in wild-type embryos (Fig. 5a, b). Loss of *Lmx1b* reduced *Bmp4* expression in the medial mesenchyme but did not alter expression of this gene in the DMe or lateral mesenchyme (Fig. 5b, d, e; Supplementary Fig. 14). Based on in situ hybridization, loss of *Lmx1a* did not compromise *Bmp4* expression in either compartment (Fig. 5b, c, e; Supplementary Fig. 14). Although in situ hybridization is well suited for spatial distribution of gene expression, it is not strictly quantitative. We, therefore, used qRT-PCR analysis of laser capture microdissected dorsal mesenchyme and DMe to more quantitatively assess expression of *Bmp* genes in each compartment (Fig. 5a, f; Supplementary Fig. 15). This analysis revealed that loss of *Lmx1a* decreased expression levels of both *Bmp4* and related *Bmp2* in the DMe. It also confirmed that loss of *Lmx1b* alone caused drastic decrease of *Bmp4* as well as *Bmp2* expression in medial mesenchyme. In double mutants, *Bmp4/2* mesenchymal expression levels were similar to those in *Lmx1b* mutants, while double-mutant DMe *Bmp4/2* expression levels were similar to those in *Lmx1a* mutants (Fig. 5f; Supplementary Fig. 15). A definitive readout of Bmp signal reception, pSmad immunostaining intensity^33^, was significantly decreased in the DMe of double but not single-gene mutants at e10.5 (Fig. 5g–k).

**Fig. 6:**
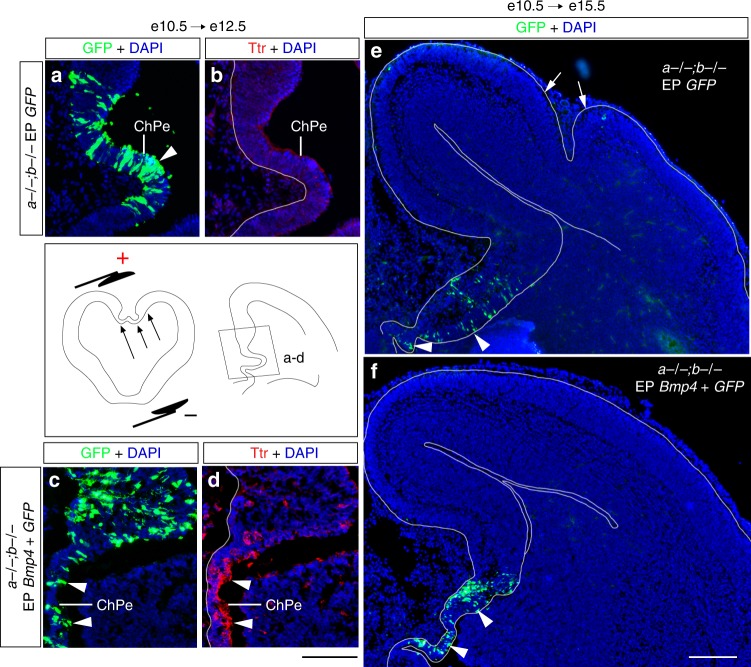
Bmp4 rescues DMe development and cortical gyrification in *Lmx1a−/−;b−/−* mutants. *Bmp4* and/or *GFP* expressing plasmids were electroporated into DMe of *Lmx1a−/−;b−/−* embryos at e10.5 and analyzed at e12.5 (**a**–**d**) or e15.5 (**e**, **f**). To electroporate DMe, the positive electrode was placed against this region, as shown in the schematic below panel **a**. Negatively charged DNA moves toward the positive electrode (arrows) entering DMe. **a**–**d** Show DMe (a region boxed in the diagram below panel **b**). Immunohistochemistry revealed that *Bmp4*, but not *GFP*, induced Ttr in the *Lmx1a−/−;b−/−* ChPe (arrowheads in **d**). GFP+ ChPe cells (arrowheads in **a**, **c**) confirm successful electroporation of ChPe. **e**, **f** While *GFP*-electroporated *Lmx1a−/−;b−/−* mutants had cortical gyrification at e15.5 (**e**, arrows point to cortical gyri), electroporation of *Bmp4* into DMe restored cortical lissencephaly (**f**). Arrowheads point to GFP+ cells in the DMe, showing specific electroporation of this domain. Scale bars: 100 μm (**a**–**d**); 200 μm (**e**, **f**)

We hypothesized that dual loss of *Lmx1a* and *Lmx1b* reduced early Bmp signaling levels below a critical threshold required to pattern and maintain normal DMe and hence subsequent lateral cortical development. We, therefore, selectively electroporated *Bmp4* into the DMe territory of *Lmx1a−/−;b−/−* embryos at e10.5. At e12.5 the double mutant DMe was rescued as demonstrated by the reappearance of *Ttr* (Fig. 6a–d; Supplementary Fig. 16a, b) and reduction of abnormally high proliferation in the ChPe (Supplementary Fig. 17). Ectopic *Wnt3a* expression in the double-mutant ChPe was also lost and low levels of Wnt signaling were restored in the distal cortical primordium as revealed by analysis of expression of downstream Wnt-β-catenin target gene *Axin2* (Supplementary Fig. 16). At e15.5, the cortical plate of electroporated *Lmx1a−/−;b−/−* mutants was lissencephalic, with no evidence of gyrification (Fig. 6e, f). Thus, supplementing Bmp at the DMe was sufficient to normalize DMe development and Wnt signaling and suppress gyrification of the distantly located cortex in *Lmx1a−/−;b−/−* embryos. We conclude that *Lmx1a* and *Lmx1b* regulate DMe development via Bmp signaling and that misregulation of the DMe development and its Wnt signaling is central to the *Lmx1a−/−;b−/−* cortical gyrification phenotype.

## Discussion

**Fig. 7:**
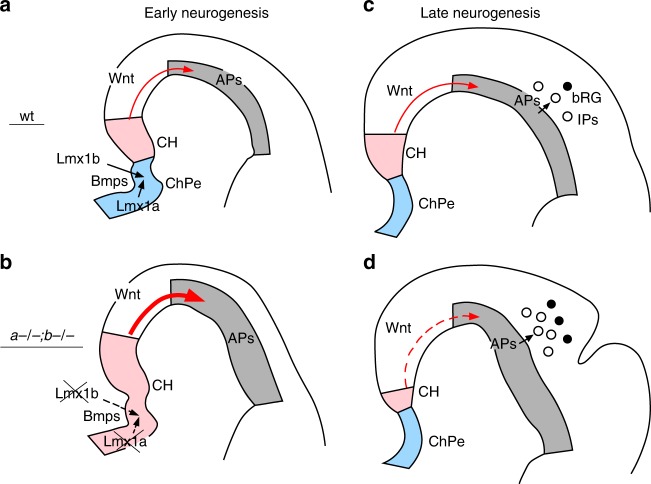
*Lmx1a/b*-dependent dorsomedial tissue interactions regulate cortical gyrification. **a** In early wild-type embryos, *Lmx1a* and *Lmx1b* are expressed in the telencephalic DMe and adjacent mesenchyme, respectively, and promote expression of secreted Bmps within their respective territories. Appropriate level of Bmp signaling specifies ChPe and CH in the DMe. Wnt3a signaling from the CH (red arrow) promotes proliferation of APs in the cortical VZ. Although we show Wnt signaling by arrow extending through neuroepithelium, our data cannot exclude a possibility that Wnt molecules are delivered to neocortical primordium via CSF. **b** Simultaneous loss of *Lmx1a* and *1b* reduces *Bmp* expression in both the mesenchyme and DMe, decreasing the level of Bmp signaling in the DMe. This results in DMe overproliferation and mispatterning, creating an enlarged *Wnt3a* expression domain at the DM in early *Lmx1a−/−;b−/−* embryos. The excessive secreted Wnt3a (large red arrow) overactivates canonical Wnt-β-catenin signaling in distal cortical primordium, leading to excessive expansion of APs in *Lmx1a−/−;b−/−* mutants. **c** At later developmental stages, in wild-type embryos, Wnt-β-catenin signaling (red arrow) limits production of IPs and bRG from APs^29^. **d** By e15.5, normal DMe specification is restored in *Lmx1a−/−;b−/−* embryos, but enhanced apoptosis reduces the size of CH and DM-derived Wnt signaling (dashed red line). Reduced Wnt signaling allows excessive production of IPs and bRG from an earlier expanded AP population, resulting in cortical gyrification

Wnt signaling from medially located CH induces hippocampus and regulates cortical size, while mesenchymal signaling regulates proliferation of cortical APs and differentiation and migration of a subset of cortical neurons^40–46^. Here we show that simultaneous loss of two transcription factors, Lmx1a, expressed in the telencephalic DMe, and Lmx1b, expressed in head mesenchyme, causes folding of the mouse cerebral cortex, implicating head mesenchyme and DMe in the regulation of cortical gyrification. Our data demonstrate that *Lmx1a* and *Lmx1b* normally act to suppress cortical gyrification in the mouse via Bmp-dependent regulation of DMe development and its Wnt signaling dynamics (Fig. 7).

Loss of *Lmx1a* and *1b* reduced expression of genes encoding secreted Bmp4/2 molecules in the DMe and adjacent mesenchyme, respectively, decreasing overall levels of Bmp signaling in the DMe of *Lmx1a−/−;b−/−* mutants. It has been previously shown that complete loss of Bmp signaling in *Bmp receptor 1a* and *1b* double-mutant mice resulted in failure to induce both the ChPe and CH^34^. Reduced Bmp signaling in *Lmx1a/b* double mutant, however, resulted in a qualitatively different phenotype. Specifically, we observed initial mispatterning of the DMe with delayed differentiation of the ChPe and expansion of *Wnt3a* expression across the prospective ChPe territory. Later during development, contraction of the *Wnt3a*+ CH domain occurred as apoptosis was elevated and a delayed *Ttr*+ ChPe developed in the *Lmx1a−/−;b−/−* DMe (Fig. 7). Exogenous medial Bmp4 rescued DMe patterning in *Lmx1a−/−;b−/−* embryos, directly implicating downregulated Bmp signaling in the mutant phenotype. Thus, our studies demonstrate that DMe development is particularly sensitive to the level of Bmp signaling in vivo. Previous in vitro analysis revealed that high Bmp levels are needed to induce the ChPe fate^47^. While low Bmp signaling in *Lmx1a−/−;b−/−* mutants was sufficient to induce *Wnt3a*+ CH by e12.5, delayed differentiation of ChPe until later developmental stages potentially reflects a delay in accumulation of threshold levels of Bmp signaling necessary for ChPe development. Regardless, our study revealed that Bmp signaling is required not only for the initial induction of the CH but also regulates its size and, thus, the temporal dynamics of DMe-derived Wnt3a signaling throughout development. Additionally, we identify that Bmp proteins produced both locally in the DMe and in the adjacent mesenchyme participate redundantly in DMe development.

Expansion of BPs and subsequent production of neurons from these progenitors has been identified as a major driving force of cortical gyrification in naturally gyrencephalic mammals. During development, BPs are generated from APs, and a typical neurogenic sequence in gyrencephalic mammals includes initial expansion of APs followed by an extensive production of bRG and IPs during later peak of neurogenesis^3,8–12^. Expansion of APs in the VZ, although necessary for BPs production, does not automatically lead to excessive generation of BPs, which need to delaminate from the VZ^4,11,48^. In the cortical VZ, APs expand via symmetric divisions, while asymmetric divisions of APs result in the production of BPs^11,29^.

Wnt/β-catenin signaling expands APs by promoting symmetric divisions and inhibiting asymmetric divisions, at least partially by controlling mitotic spindle orientation^29^. In contrast, reduction of Wnt signaling favors asymmetric cell divisions, activating the production of BPs (both bRG and IPs) from APs, depleting the AP pool^29,39^. Wnt/β-catenin signaling, however, has not previously been considered a regulator of cortical gyrification because neither constitutive gain-of-function nor loss-of-function Wnt/β-catenin mouse models showed bona fide cortical gyrification^3,11,29,31,39^. Constitutive overexpression of a transgene encoding stabilized β-catenin in neural precursors produced a *pseudo*-gyrified cortex with both the ventricular and pial surfaces equally folded^31^, rather than the asymmetric increase in pial surface as seen in naturally gyrencephalic mammals^3^. Additionally, there was a profound loss of cortical lamination in these transgenic mice^31,49^. Further, mice with permanently activated Wnt/β-catenin signaling demonstrated a decrease in the number of BPs, rather than an increase as observed in higher mammals^39^.

Early constitutive ablation of β-catenin in the developing mouse telencephalon increased the numbers of both bRG and IPs but was insufficient to induce gyrification^29,39^. Although both bRG and IPs were increased in the number in the telencephalon of β-catenin ablated mice, IPs were expanded only at early stages likely because by the peak of neurogenesis, APs were severely depleted and could not further support the generation of excessive IPs^29^.

In contrast, in *Lmx1a−/−;b−/−* mutants, reduction of Wnt/β-catenin signaling occurred only after its transient overactivation and the resultant overexpansion of AP pool at earlier stages. When Wnt signaling was reduced at e15.5, at the peak of cortical neurogenesis, this expanded AP population was apparently sufficient to simultaneously produce enough bRG and IPs to drive cortical gyrification. Consistently with this hypothesis, suppression of the early activation of Wnt/β-catenin signaling by electroporating Wnt inhibitor *Dkk1* into the cerebral cortex, reduced the number of cortical APs, bRG and IPs and restored lissencephaly in *Lmx1a−/−;b−/−* mice, revealing a critical role of Wnt signaling in the development of cortical gyrification in this mutant (Fig. 4).

Interestingly, despite local overexpansion of BPs in the emerging gyri in e15.5 *Lmx1a−/−;b−/−* embryos, we could not identify differences in progenitor behavior in different cortical regions in e12.5 *Lmx1a−/−;b−/−* mice. Thus, location of gyri and sulci in *Lmx1a−/−;b−/−* mice is likely determined by region-specific responses of initially expanded APs to reduced Wnt signaling at later stages, possibly resulting in differential delamination and/or migration of BPs. The molecular basis of differential response of different cortical regions to secreted factors remains unknown. Notably, however, we have recently reported that in another cortical gyrification mouse model, subtle changes in polarity at the apical ventricular surface coincide with location of gyri and are accompanied by localized accumulation of nuclear YAP, which is essential to drive localized proliferation of BPs^21^.

In the canonical Wnt pathway, Wnt ligands bind to LRP5/Frizzled receptors leading to translocation of β-catenin to the nucleus and activation of transcription of Wnt-β-catenin responsive genes^50^. In *Lmx1a−/−;b−/−* mutants, the rate of change of Wnt signaling, as revealed by expression levels of canonical Wnt pathway downstream genes *Lef1* and *Axin2*, was fast: the switch from high to low Wnt signaling happened within 3 days (from e12.5 to e15.5), leading to dramatic changes in cortical progenitor populations. Fast effects of misregulated Wnt signaling have also been described in other experimental systems. For example, activating or reducing activity of Wnt pathway resulted in dramatic biological responses in wild-type chicken spinal cord or mouse neocortex already 24 h after electroporation of expression plasmids^39,51^ highlighting that tight regulation of Wnt signaling is critical for normal development of the central nervous system. Analysis of *Lef1* expression or an *Axin2* reporter revealed a Wnt signaling gradient that in the wild-type telencephalon extends through neuroepithelium from the DMe (high) to lateral neocortex (low)^40,52^ (Supplementary Fig. 11a). The existence of a Wnt response gradient through the cortical neuroepithelium suggests that DMe-derived Wnt ligands diffuse (or are actively transported) via neuroepithelium. In e12.5 *Lmx1a−/−b−/−* mutants, a medio-lateral *Lef1* expression gradient was still observed; however, it was brighter than in comparably staged wild-type controls (Supplementary Fig. 11a, b), as expected by the presence of an enlarged *Wnt3a*-expressing DMe domain. Previously, several Wnts have been also detected in mouse and human cerebrospinal fluid (CSF)^53,54^. Thus, we cannot exclude a possibility that in *Lmx1a−/−;b−/−* mutants, some Wnt molecules reach neocortex via CSF as well.

Importantly, rescue of the DMe patterning in *Lmx1a−/−;b−/−* mutants by early electroporation of *Bmp4* into the DMe was sufficient to normalize activity of the Wnt-β-catenin signaling in the distantly located neocortex to restore lissencephaly. These data confirm that the DMe was the primary source of a gyrification-related Wnt signaling in *Lmx1a−/−;b−/−* mutants. Our experiments, however, do not exclude a possibility that other signaling molecules, beyond Wnt3a from the DMe, contribute to cortical gyrification in *Lmx1a/b* double mutants. For example, signaling from lateral mesenchyme located adjacent to the neocortical primordium may directly contribute to cortical developmental abnormalities in *Lmx1a/b* mutants. However, we consider this an unlikely possibility since cortical gyrification was not detected in *Lmx1b−/−* mutants. Thus, even if *Lmx1b*-dependent signaling from lateral mesenchyme contributes to cortical gyrification in *Lmx1a−/−;b−/−* mutants, it still does so in cooperation with the DMe-derived Wnt signaling, which is dependent on both *Lmx1* genes. Therefore, our experiments combining *Lmx1a/b* mutants analysis with *Bmp4/Dkk1* electroporation studies identify misregulation of DMe-derived Wnt signaling as a major contributor to the cortical phenotype of *Lmx1a−/−;b−/−* mutants, identifying a mesenchymal/DMe Lmx1a/1b-Bmp-Wnt pathway as a regulator of cortical gyrification.

Our study clearly demonstrates that lissencephaly in mice is maintained by redundant function of two *Lmx1* genes. Genetic redundancy is believed to be maintained in evolution to stabilize key developmental processes in animal populations to make them less susceptible to various influences such as environmental conditions or genetic background^55^. Given the important role of DMe for cortical organization and the profound effect of relatively subtle variations of Bmp signaling on DMe development, it seems logical that the development of this particular tissue is stabilized via redundant pathways. Notably, the only two other genes known to maintain lissencephaly in the mouse (or any other lissencephalic species) are *Flrt1* and *Flrt3* which redundantly regulate neuronal adhesion during migration^25^. We conclude that lissencephaly is actively regulated during development and protected by redundant mechanisms. Disruption of these mechanisms is sufficient to induce gyrification of normally lissencephalic mouse neocortex, revealing striking plasticity of neocortical development in mice.

Similar to the developing neocortex of naturally gyrencephalic mammals, we observed local expansion of BPs in the emerging gyri but did not detect specific increases in apoptosis in cortical regions developing sulci in e15.5 *Lmx1a−/−;b−/−* embryos. Taken together, these results suggest localized expansion of BPs as a major mechanism of cortical gyrification in *Lmx1a−/−;b−/−* mutants, although we cannot exclude a possibility that transient region-specific misregulation of apoptosis, for example at earlier stages, also contributes to the phenotype. In higher mammals, cortical gyrification is a complex process that beyond localized overexpansion of BPs, involves other mechanisms as well, such as region-specific differences in neuronal migration^2–4,25^. It remains to be investigated whether other cellular mechanisms that regulate cortical gyrification in higher mammals also contribute to cortical folding in *Lmx1a−/−;b−/−* mutants.

In conclusion, we have identified a redundant Lmx1a/b-Bmp-dependent pathway that shapes the dynamics of dorsal midline Wnt signaling. In mice, this pathway operates to prevent differential expansion of cortical progenitors and suppress gyrification of the distant neocortex. It has been recently demonstrated that similar to the mouse, a Wnt3a-expressing signaling center is also present in the DMe of the naturally gyrencephalic ferret^56^. Although the mechanisms that regulate DMe development and signaling in gyrencephalic mammals remain unknown, in the developing human neocortex, expression of Wnt receptor *FZD8* is higher than in the mouse and even the chimpanzee, potentially making the developing human neocortex particularly sensitive to dynamics of Wnt signaling^57^. An important next step will be to determine whether modification of the DMe/mesenchymal signaling pathways that we describe here promote gyrification during evolution of naturally gyrencephalic mammals.

## Methods

### Mice

*Lmx1a*-null (*Lmx1a*^*drJ*^, The Jackson Laboratory strain #000636)^58^, *Lmx1b*-null (*Lmx1b−/−*)^59^, *Lmx1b*^*LacZ*^^60^, *Lmx1a-Cre*^61^, and *ROSA26-YFP*^62^ (The Jackson Laboratory strain #006148) mice were used in this study. Mice were maintained on a mixed genetic background comprising C57Bl6, 129, FVB, and CD1. Noon of the day of the plug was considered e0.5. Embryos of both sexes were used for experiments. For cell proliferation experiments, pregnant females were intraperitoneally injected with BrdU at 50 mg/kg. All animal experiments were performed in accordance with the protocols approved by the Institutional Animal Care and Use Committees of the University of Tennessee Health Science Center (UTHSC), Seattle Children’s Research Institute or the University of Chicago.

### Tissue analysis

Immunohistochemistry was performed using cryosections^63^. Embryos were collected in cold phosphate-buffered saline (PBS). Whole heads or dissected brains were fixed in freshly prepared 4% paraformaldehyde (PFA) in PBS for 1–12 h., washed three times in PBS (30 min each), equilibrated in 30% sucrose in PBS for 2 h at 4 °C, and embedded in optimum cutting temperature (OCT) compound (Tissue Tek). OCT blocks were sectioned on a cryostat at 12 μm. Sections were collected on Superfrost Plus glass slides (Fisher Scientific) and kept at −20 °C. Then, sections were dried at room temperature for 20 min, washed in PBS, blocked in PBS containing 1% normal goat serum and 0.1% Triton X-100, and incubated with primary antibodies overnight at 4 °C. Secondary antibodies were applied at room temperature for 1 h. Some sections were counterstained with DAPI (Sigma-Aldrich) to visualize tissue. Sections were mounted in Fluorogel (EMS) and coverslipped. For BrdU and Ki67 immunostaining, slides were boiled in 1× target retrieval solution (Dako) for 10 min before the blocking step. For pSmad immunostaining, slides were incubated in the same solution for 7 min at 95 °C. The following primary antibodies were used: anti-Lmx1a (goat, Santa-Cruz, catalog #sc-54273, 1:500, *RRID*:AB_2136830), anti-Cux1 (rabbit, Santa-Cruz, catalog #sc-13024, 1:200, RRID:AB_2261231), anti-Ctip2 (rat, Abcam, catalog #ab18465, 1:500, RRID:AB_2064130), anti-GFP antibody that recognizes YFP (chicken, catalog #ab13970 Abcam, 1:500, RRID:AB_300798), anti-Reelin (mouse, Chemicon, catalog #Mab5364, 1:500, RRID:AB_2179313), anti-BrdU (rat, Abcam, catalog #ab6326, 1:50, RRID:AB_305426), anti-BrdU (mouse, Rockland, catalog #600-401-c29, 1:300, RRID:AB_10893609), anti-Ki67 (mouse, BD Pharmingem, catalog #556003, 1:250, RRID:AB_396287), anti-Pax6 (rabbit, Covance, catalog #Prb-278p-100, 1:300, RRID:AB_291612), anti-Tbr2 (rat, eBioscience, catalog #14-487582, 1:200, RRID:AB_11042577), anti-β-catenin (mouse, BD Biosciences, catalog #610153, 1:200, RRID:AB_397554), anti-Lef1 (rabbit, Cell Signaling Technology, catalog #2230S, 1:300), anti-pSmad (rabbit, Chemicon, catalog #AB3848, 1:50, RRID:AB_177439), anti-Ttr (rabbit, Dako, catalog #A0002, 1:200, RRID:AB_2335696), anti-activated Caspase 3 (rabbit, Promega, catalog #G7481, 1:250, RRID:AB_430875), and anti-p73 (mouse, Fisher, catalog #MA5-14117, 1:100, RRID:AB_10987160) with species-specific secondary antibodies conjugated with Alexa 350, 488, 568, or 594 fluorophores (Life Technologies).

For Nissl staining, sections were incubated in 0.1% cresyl violet solution for 10 min, dehydrated through an ethanol/xylens series and coverslipped with Permount (Fisher)^64^. In situ hybridization was performed using digoxigenin-labeled *Lmx1a*^26^, *Lmx1b*^26^, *Bmp4*^65^, *Ttr*^66^, and *Wnt3a*^32^ RNA probes and anti-digoxigenin antibody conjugated with alkaline phosphatase (Roche). Hybridizations were performed in a buffer containing 50% formamide, 4× saline sodium citrate, and 1% sodium dodecyl sulfate at 70 °C overnight. Color reactions were carried out using NBT/BCIP substrates (4-nitroblue tetrazolium chloride/5-bromo-4-chloro-3-idolyl phosphate)^67^. X-gal staining was performed using sections from fresh-frozen embryos^68^. Slides were fixed in 0.2% glutaraldehyde in PBS for 10 min on ice and after brief rinsing were incubated in X-gal staining solution (0.02% Igepal, 0.01% sodium deoxycholate, 5 mM potassium ferricyanide, 5 mM potassium ferrocyanide, and 2 mM MgCl_2_ diluted in 0.1 M phosphate buffer) at 37 °C overnight in the dark. To visualize tissue, X-gal-stained sections were counterstained with Nuclear Fast Red (Sigma).

Images of immunostained sections (except β-catenin-stained sections) were taken using a Zeiss AxioImager A2 microscope equipped with an AxioCam Mrm camera and AxioVision Rel 4.9 software (Zeiss). Images of β-catenin-immunostained sections were taken using a Zeiss LCM 710 laser scanning confocal microscope with Zen software. Brightfield images were taken using a Leica MZFLIII microscope with Leica DFC425 camera and LAS V3.8 software or Olympus SZX microscope with Leica MC170HD camera and LAS V4.5 software. Figure panels were assembled in Photoshop CC 2018 (Adobe).

### Mouse in utero electroporation

Plasmids encoding GFP, Dkk1, and Bmp4 (1 μg/μl) were injected using a fine-glass microcapillary into a lateral ventricle and electroporated into either telencephalic DMe or the neocortical primordium using paddle electrodes with BTX ECM830 electroporator^69^. To target the telencephalic DMe, the positive electrode was placed adjacent to the DMe, as shown in Fig. 6 (in schematic below Fig. 6a). The neocortical primordium was targeted by placing the positive electrode against the neocortical wall. To facilitate injections, Fast Green dye was added to plasmid solutions. The following plasmids were used: *pCIG*-mouse *Dkk1*^39^, *pCAGGS*-mouse *Bmp4*^70^, and *pCAGGS-GFP*^71^. After electroporation, the uterus was returned to abdomen, and the embryos continued development. Upon collection, embryos were evaluated under a fluorescence stereo microscope, and those showing GFP fluorescence in appropriate brain regions were serially sectioned on a cryostat and used for analysis.

### Laser capture microdissection (LCM) and qRT-PCR

Embryonic heads were flash-frozen in OCT on dry ice and coronally sectioned on a cryostat at 10 μm. Sections for LCM were mounted on uncharged glass slides (AmLabs^TM^), dried at room temperature for 30 s, and stained with hematoxylin and eosin to visualize tissue morphology, using HistoGene LCM Frozen Sections Staining kit (Fisher Scientific, Cat #KIT0401)^72^. LCM was performed using an ArcturusXT laser capture microdissection machine with Arcturus CapSure Macro LCM caps. For *Bmp* expression analysis, five telencephalic sections separated from each other by 10 μm were used for LCM from each e10.5 embryo. Adjacent sections were immunostained against Lmx1a and used as templates to localize boundaries of the telencephalic DMe. For analysis of mesenchyme, mesenchymal tissue located above the DMe was microdissected (green domain in Fig. 5a). For *Axin2* expression analysis in e12.5 and e15.5 embryos, cortical primordium located between the dorsal cortical bend and LGE (gray region in schematics adjacent to Supplementary Fig. 11c and Supplementary Fig. 12b) was microdissected from 4 to 6 non-adjacent telencephalic sections from each embryo. For gene expression analysis in Bmp rescue experiments (Supplementary Fig. 16), sections of *pCAGGS-Bmp4* and/or *pCAGGS-GFP*-electroporated embryos were first evaluated under a fluorescence microscope and embryos that specifically expressed GFP in the DMe (similar to those shown in Fig. 6e, f) were identified. ChPe and neocortical primordium from sections from these embryos were isolated by LCM and used for gene expression analysis.

RNA from microdissected samples was isolated using Pico Pure RNA purification kit (Arcturus KIT#0204). Before eluting RNA, RNAse free DNAse I (Qiagen) was applied to each column and incubated for 15 min at room temperature to remove traces of genomic DNA. cDNA was generated using cDNA synthesis kit (Biorad, cat#1708890). qRT-PCR was performed on Roche LC480 Real-time PCR machine with SYBR Fast qPCR master mix (Kapa Biosystems)^72,73^. Transcript levels were normalized to *Gapdh*. All samples were tested in triplicates. At least three embryos per genotype per experimental condition were tested using primers listed in Supplementary Table 1.

### Cell counts, measurements, and statistical analysis

For each embryo, the telencephalon was coronally serially sectioned and the same number of sections was placed on each slide to identify the position of each section along the anterior–posterior (A–P) axis of the telencephalon. For consistency, all comparisons between mutant and control embryos were performed using sections taken at similar A–P levels, approximately midway along the A–P axis of the telencephalon. At least three embryos per genotype or experimental condition (typically three non-adjacent sections per embryo) were evaluated in each analysis at each developmental stage.

Cortical parameters (area, regional thickness of cortical neuroepithelium) were measured between the telencephalic dorsal bend (pointed by upper arrow in Supplementary Fig. 1e) and LGE (pallial-subpallial boundary) (lower arrow in Supplementary Fig. 1e)^74–76^, using ImageJ (NIH). For each embryo, cortical area in left and right hemispheres was added to generate a total cortical area, which was compared between *Lmx1a−/−;b−/−* and control littermates (Supplementary Fig. 1f). For clarity, cortical area in *Lmx1a−/−;b−/−* mutants was normalized to that in wild-type controls. To evaluate whether cortical gyri occupy non-random positions or are randomly distributed throughout e18.5 *Lmx1a−/−;b−/−* neocortex, the cortical ventricular surface (between the dorsal cortical bend and LGE) was divided into 10 equally sized segments (Supplementary Fig. 1e). Cortical thickness was measured in the middle of each segment in different embryos and plotted (Supplementary Fig. 1g, h). Cortical progenitors were counted in 100-μm-wide radial segments (columns) spanning the entire thickness of the neocortex, from ventricular to outer cortical surface. For consistency, in *Lmx1a−/−;b−/−* embryos, cell counts were performed in dorsal neocortical primordium, which develops a dorsal bulge, the most consistent cortical gyrus in *Lmx1a−/−;b−/−* embryos (Supplementary Fig. 1b–d, h). In control embryos, cell counts were performed in the same region of the cortex as in *Lmx1a−/−;b−/−* mutants. IPs were defined as Tbr2+ cells^16,29^. Originally, bRG cells were defined as Pax6+/Tbr2− progenitors that occupy the SVZ and possess a single basal process^8,9^. More recently, however, bRG cells with diverse morphology were described, indicating that apical-only process is not a definitive feature of bRG^11,77^. Thus, similar to Wang et al.^16^ and Draganova et al.^29^, we identified bRG as Pax6+/Tbr2− cells located basal to the Tbr2+IP band. Similar to wild-type controls, in *Lmx1a−/−;b−/−* mutants, these basal Pax6+/Tbr2− cells were Ki67+ and incorporated BrdU after a short BrdU pulse (Supplementary Fig. 3a–e, g–i), indicating that they indeed were proliferating progenitors. To compare distribution of proliferating cells in e15.5 wild type and *Lmx1a−/−;b−/−* embryos (Supplementary Fig. 3k), a 90 min BrdU pulse was performed and fractions of basally located proliferating (S-phase) cells (the number of BrdU+ cells located beyond the Pax6+VZ divided by the total number of BrdU+ cells) were calculated. To evaluate activity of the Bmp signaling pathway in the telencephalic DMe of e10.5 embryos, intensity of pSmad immunostaining was measured using ImageJ (NIH)^33^. The boundaries of the DMe were identified using adjacent Lmx1a-immunostained sections as templates. To analyze cell-cycle exit, embryos were harvested 20–24 h after a BrdU pulse and the fraction of cells that exited the cell cycle (the number of BrdU+/Ki67− cells divided by the total number of BrdU+ cells) was calculated in the neocortex^78^.

Statistical analysis was performed using IBM SPSS Statistics (version 25) software. All quantitative data are presented as the mean ± s.d. Statistical analysis between two groups was performed using two-tailed *t*-test. Multiple groups were analyzed using one-way ANOVA with Tukey’s post hoc test. Bonferroni correction was applied when multiple comparisons were performed. *P* < 0.05 was considered statistically significant.

### Reporting summary

Further information on research design is available in the Nature Research Reporting Summary linked to this article.

## Supplementary information

Supplementary Information

Reporting Summary

Source Data

## Data Availability

The data that support the findings of this study are available from the corresponding authors upon reasonable request. Source data for Fig. 1k, n; 2f, i, l, o, r, u; 4f–h; 5f, k and Supplementary Figs. 1f–h; 3k–o, r; 4d; 7e; 9g, l; 11c, d; 12b, c; 15 and 16b, c are provided as a Source data file.
