# Supplementary material for: Early dorsomedial tissue interactions regulate gyrification of distal neocortex
Source: Nat Commun. 2019 Nov 15;10:5192. doi: 10.1038/s41467-019-12913-z (PMC6858446; doi:10.1038/s41467-019-12913-z)
Supplement: Supplementary file 1 — Supplementary Information [file 41467_2019_12913_MOESM1_ESM.pdf]

Supplementary Information

Early dorsomedial tissue interactions regulate gyrification of distal neocortex.

Chizhikov et al.

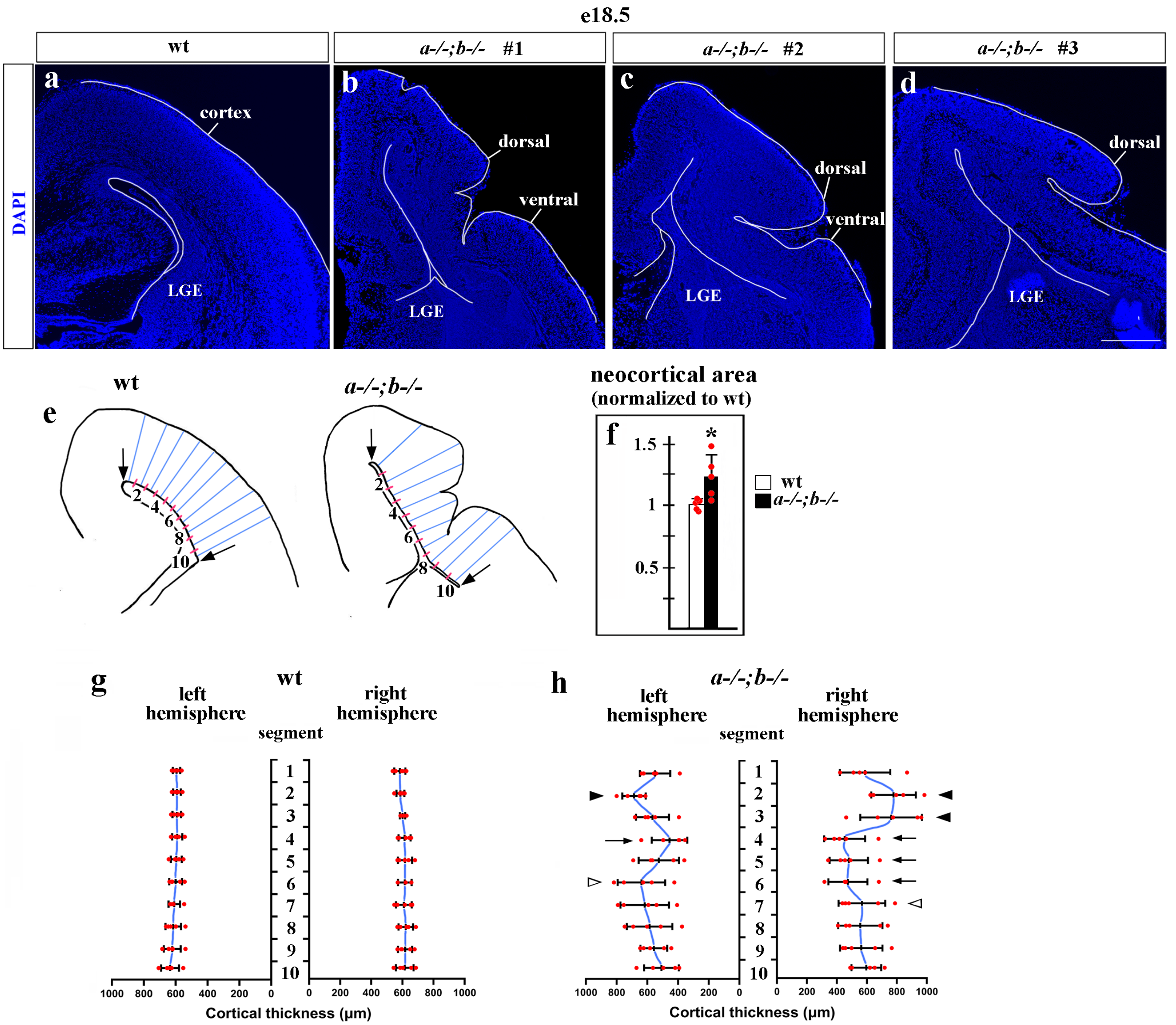

Supplementary Fig. 1. Non-random pattern of cortical gyrification in e18.5 *Lmx1a-/-;b-/-* mutants. (**a-d**) DAPI-stained right coronal hemisections from a wild-type embryo and three *Lmx1a-/-;b-/-* mutants from three independent litters at e18.5. A big dorsal neocortical gyrus (labeled as dorsal) was evident in all three *Lmx1a-/-;b-/-* embryos. Two *Lmx1a-/-;b-/-* embryos (#1 and #2) also had an additional, more ventrally located neocortical gyrus (ventral). LGE – lateral ganglionic eminence.

(**e**) Diagrams illustrating cortical measurements. The neocortical ventricular surface (between the dorsal cortical bend, upper arrow, and LGE, lower arrow) was divided into 10 equally sized segments. Cortical thickness was measured in the middle of each segment in different embryos. Cortical area was measured between the dorsal cortical bend and LGE as well.

(**f**) Cortical area was significantly higher in *Lmx1a-/-;b-/-* embryos compared to wild-type controls (two-tailed t-test, n=5 embryos per genotype, *p<0.05).

(**g, h**) Plots of regional cortical thickness in e18.5 wild-type and *Lmx1a-/-;b-/-* embryos (n=5 embryos per genotype). Connecting average cortical thickness values in neighboring regions resulted in a smooth cortical surface in wild-type embryos, but in a clearly gyrified cortical surface in *Lmx1a-/-;b-/-* mutants, illustrating non-random distribution of cortical gyri. (**h**) Arrowheads point to the dorsal gyrus that was evident in cortical segment 2 in left hemisphere and segments 2 and 3 in right hemisphere. Arrows point

to a sulcus (thinnest neuroepithelium, segment 4 in left hemisphere and segments 4-6 in right hemisphere). Open arrowheads point to less prominent ventral gyri (segment 6 in left hemisphere and segment 7 in right hemisphere).
All data are mean ± sd. Source data for panels **f-h** are provided as Source Data File. Scale bar: 400 µm.

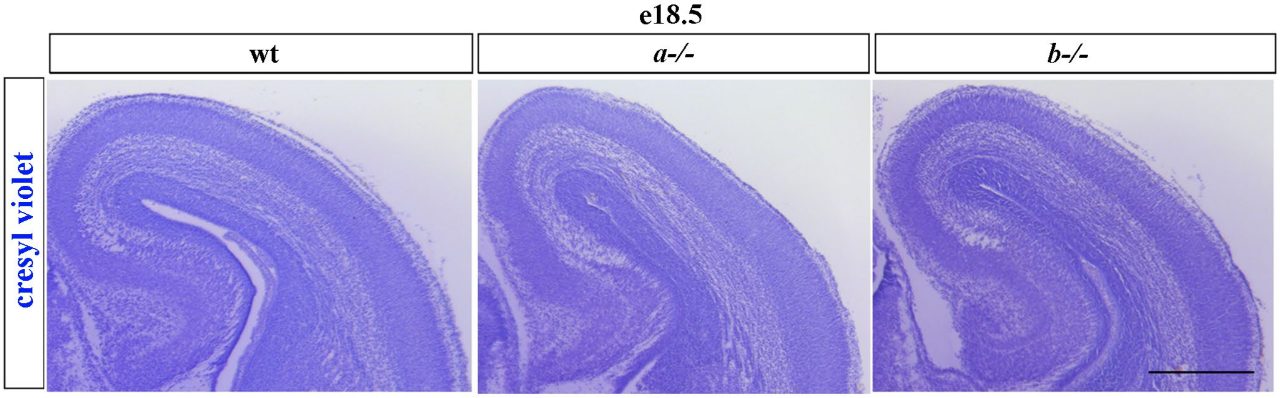

Supplementary Fig. 2. Lissencephalic cortex of *Lmx1a* and *Lmx1b* single-gene mutants.
Nissl-stained telencephalic coronal hemisections from e18.5 wild-type, *Lmx1a-/-* and *Lmx1b-/-* embryos, showing lissencephalic cerebral cortex in all three genotypes.
Scale bar: 500 µm.

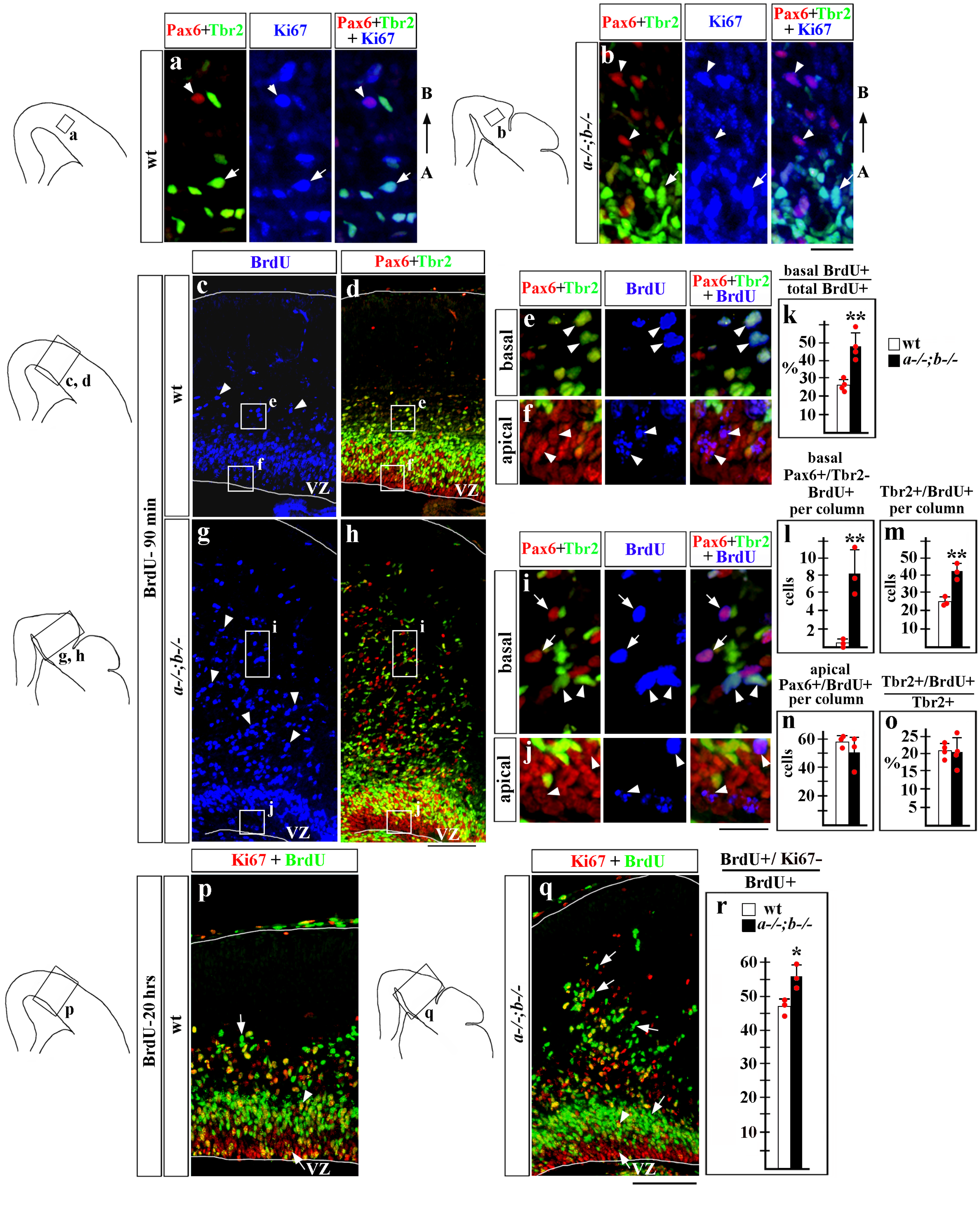
Pax6+Tbr2
Ki67
Pax6+Tbr2 + Ki67
a
b
wt
a-/-;b-/-
B
A
BrdU
c
d
e
f
g
h
i
j
VZ
BrdU- 90 min
basal
apical
Pax6+Tbr2 + BrdU
basal BrdU+ / total BrdU+
k
%
wt
a-/-;b-/-
basal Pax6+/Tbr2- BrdU+ per column
Tbr2+/BrdU+ per column
l
m
cells
apical Pax6+/BrdU+ per column
Tbr2+/BrdU+ / Tbr2+
n
o
Ki67 + BrdU
p
q
BrdU-20 hrs
BrdU+/ Ki67- / BrdU+
r

Supplementary Fig. 3. Expansion of proliferating BPs and cell cycle exit in e15.5 *Lmx1a-/-;b-/-* gyri. Coronal sections at e15.5. High magnification panels correspond to regions boxed in adjacent schematics or panels.
(**a, b**) In both wild-type and *Lmx1a-/-;b-/-* cortex, Pax6+/Tbr2- bRG (arrowheads) and Tbr2+ IPs (arrows) expressed a progenitor marker Ki67. Apical (A) is down, basal (B) is up.
(**c-j**) Mice were injected with BrdU 90 min. prior to collecting embryos. (**c, g**) Arrowheads point to basally located BrdU+ (S-phase) cells. (**k**) A fraction of basally located BrdU+ cells (the number of basally located BrdU+ cells divided by the total number of BrdU+ cells) was dramatically increased in *Lmx1a-/-;b-/-* cortical gyri (n=4 embryos per genotype, two-tailed t-test, $^{**}p<0.01$).
(**e, i**) Arrowheads point to BrdU+/Tbr2+ (S-phase) IPs, arrows point to BrdU+/Pax6+/Tbr2- (S-phase) bRG. (**l, m**) The number of both BrdU+/Pax6+/Tbr2- bRG and BrdU+/Tbr2+ IPs increased in *Lmx1a-/-;b-/-* cortical gyri related to wild-type controls (n=3 embryos per genotype, two-tailed t-test, $^{**}p<0.01$).
(**f, j**) Arrowheads point to BrdU+ (S-phase) Pax6+/Tbr2- APs in VZ. (**n**) The number of BrdU+/Pax6+ (S-phase) APs was not significantly different between cortical gyri of *Lmx1a-/-;b-/-* embryos and controls (n=3 embryos per genotype, two-tailed t-test, $p>0.05$).
(**o**) The fraction of BrdU+ (S-phase) IPs (the number of BrdU+/Tbr2+ cells divided by the total number of Tbr2+ cells) was not significantly different between cortical gyri of *Lmx1a-/-;b-/-* embryos and controls (n=4 embryos per genotype, two-tailed t-test, $p>0.05$).
(**p, q**) Mice were injected with BrdU 20 hrs. prior to collecting embryos. Arrowheads point to cells that re-entered the cell-cycle (BrdU+/Ki67+ cells), arrows – to cell that exited the cell-cycle (BrdU+/Ki67- cells). (**r**) A higher fraction of cells exited the cell-cycle (the number of BrdU+/Ki67- cells divided by the number of BrdU+ cells) in *Lmx1a-/-;b-/-* neocortical gyri compared to wild-type cortex (n=3 embryos per genotype, two-tailed t-test, $^{*}p<0.05$).
All data are mean $\pm$ sd. Source data for panels **k**, **l-o**, **r** are provided as Source Data File.
Scale bars: 100 μm (**c, d, g, h, p, q**), 25 μm (**a, b, e, f, i, j**).

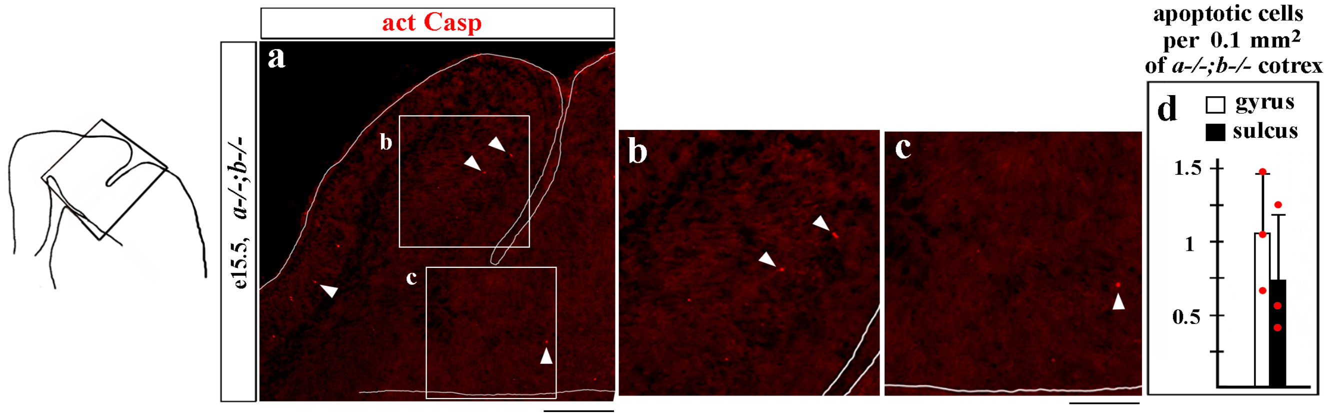

Supplementary Fig. 4. Apoptosis is not elevated in sulci-forming regions of *Lmx1a-/-;b-/-* neocortex. Panel **a** corresponds to the region boxed in adjacent diagram. Panels **b** and **c** show higher magnification of neocortical regions developing a gyrus and a sulcus, respectively. Arrowheads point to Caspase 3+ (apoptotic) cells. (**d**) There was no increase in the number of apoptotic cells per area in neocortical regions developing sulci relative to those developing gyri in *Lmx1a-/-;b-/-* neocortex ($p>0.05$, two-tailed t-test, n=3 embryos), indicating that increased localized cell death in sulci-forming regions is not a major cause of cortical gyrification in *Lmx1a-/-;b-/-* mutants.
All data are mean $\pm$ sd. Source data for panel **d** is provided as Source Data File.
Scale bars: 200 μm (**a**); 100 μm (**b, c**).

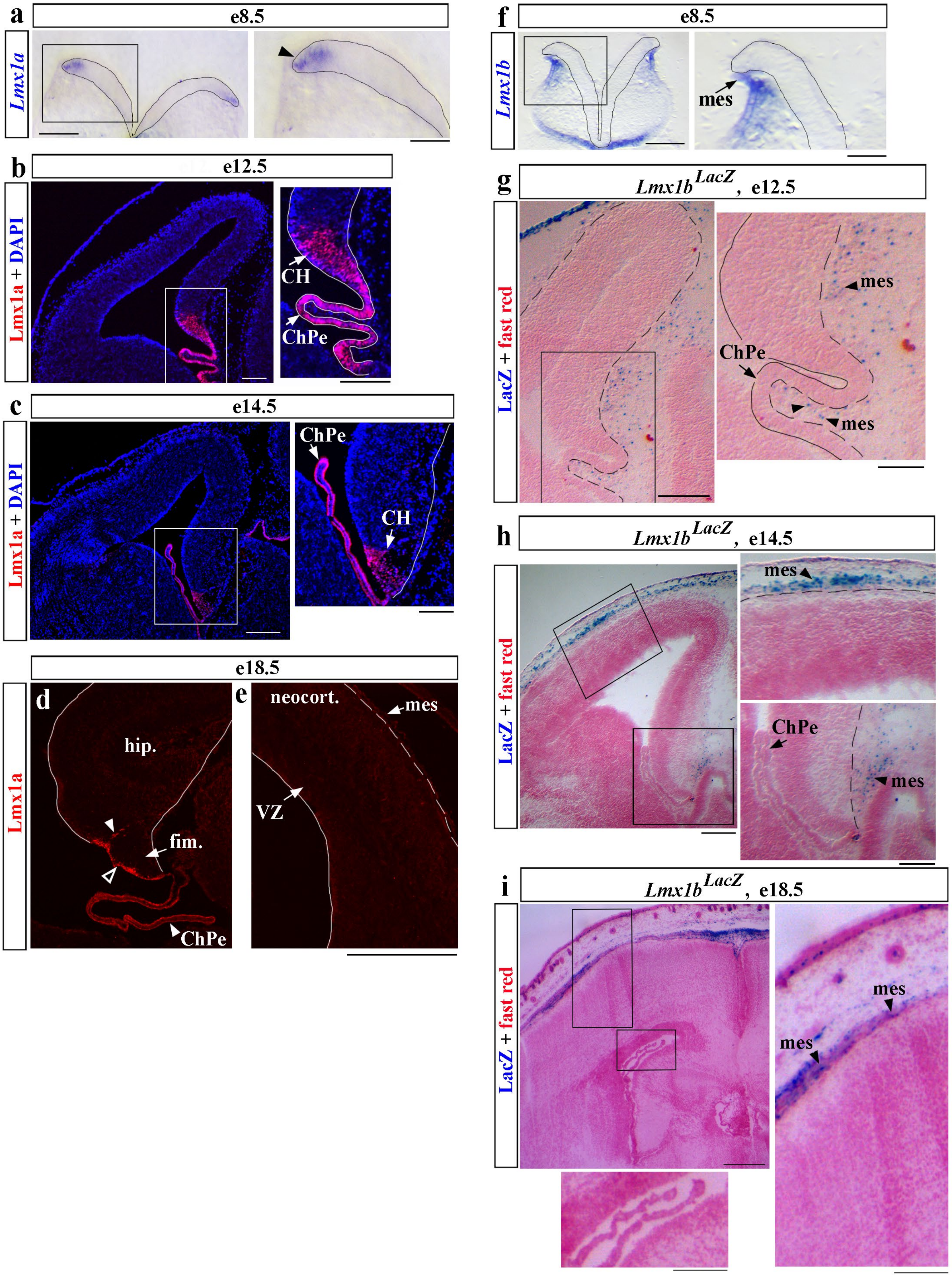

a
e8.5
Lmx1a
b
e12.5
Lmx1a + DAPI
CH
ChPe
c
e14.5
Lmx1a + DAPI
ChPe
CH
e18.5
Lmx1a
d
hip.
fim.
ChPe
e
neocort.
mes
VZ
f
e8.5
Lmx1b
mes
g
Lmx1b^LacZ, e12.5
LacZ + fast red
mes
ChPe
mes
h
Lmx1b^LacZ, e14.5
LacZ + fast red
mes
ChPe
mes
i
Lmx1b^LacZ, e18.5
LacZ + fast red
mes
mes

Supplementary Fig. 5. Complementary expression of *Lmx1a/b* in the developing telencephalon. Coronal telencephalic sections of wild-type (**a-f**) or *Lmx1b*^{LacZ} (**g-i**) embryos stained with *Lmx1a* or *Lmx1b in situ* probes (**a, f**), anti-Lmx1a antibodies (**b-e**) or for β-gal activity (**g-i**). Sections **b-c** and **g-i** were counterstained with DAPI or nuclear fast red, respectively. High magnification panels correspond to regions boxed in adjacent lower magnification panels.

(**a**) At e8.5, *Lmx1a* was expressed in lateral folds (arrowhead) of the closing anterior neural plate. (**b-c**) At e12.5-14.5, Lmx1a was specifically expressed in the ChPe and CH.

(**d, e**) At e18.5, Lmx1a was expressed in the ChPe, in a thin layer of cells populating ventricular surface of fimbria (fim.) (**d**, open arrowhead), which is the derivative of the CH, and in a few deeper located cells at the fimbria/hippocampus border (**d**, arrowhead). No Lmx1a expression was detected in the cerebral cortex or mesenchyme (**a-e**).

(**f**) At e8.5, *Lmx1b* was expressed in mesenchyme (mes.) adjacent to lateral folds (arrow).

(**g**) In e12.5 *Lmx1b*^{LacZ} embryos, β-gal was limited to mesenchyme (arrowheads), including that located adjacent to the ChPe.

(**h**) In e14.5 *Lmx1b*^{LacZ} embryos, β-gal was limited to mesenchyme, including that located adjacent to the cerebral cortex (**upper inset**, arrowhead) and CH/hippocampus (**lower inset**, arrowhead)

(**i**) At e18.5, β-gal signal was strong in mesenchyme adjacent to neocortex (**right inset**, arrowheads) but was not detected in mesenchyme adjacent to ChPe (**left inset**).

No β -gal signal was detected in the ChPe or cerebral cortex at any stage (**g-i**).

Scale bars: 50 μm (**insets** in **panels a, f, g**); 100 μm (**a, b, f, g,** and **insets** in **panels b, c, h, i**); 500 μm (**d, e**); 200 μm (**c, h**); 250 μm (**i**).

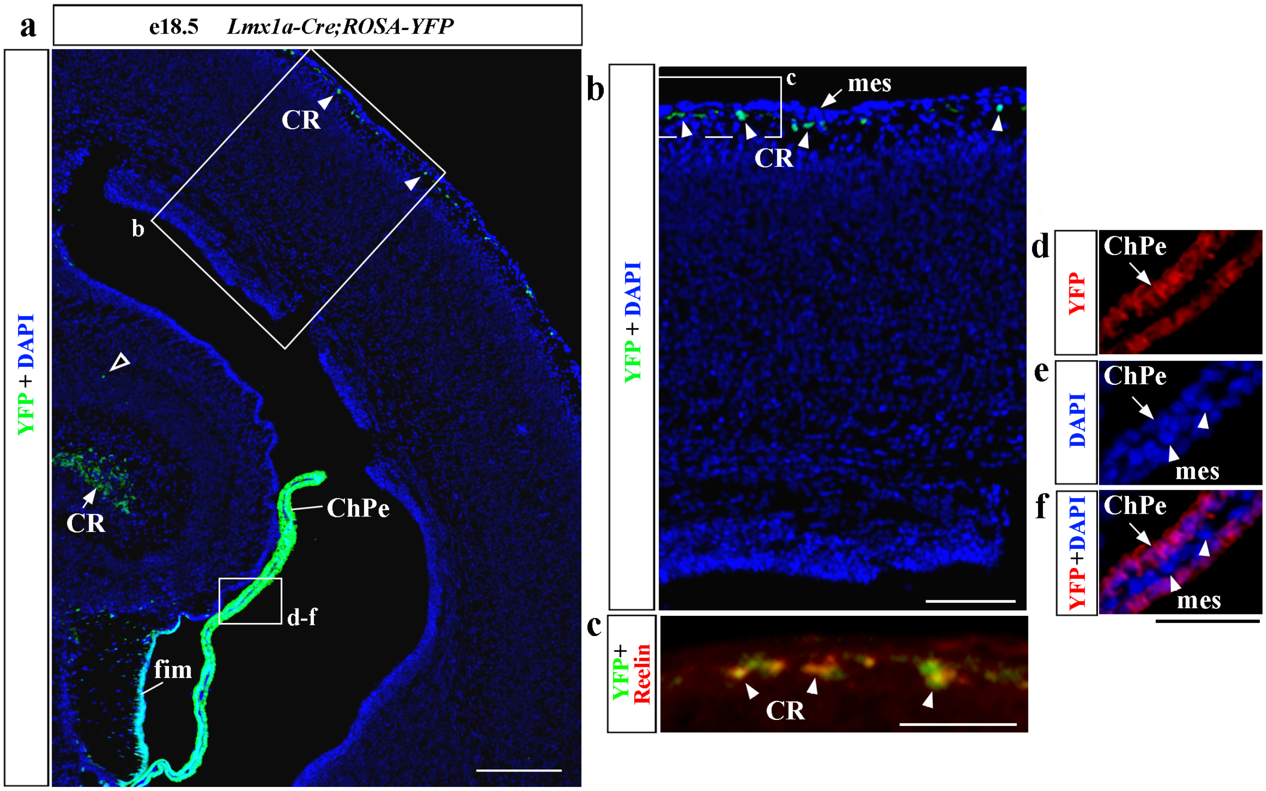

Supplementary Fig. 6. Genetic fate mapping to identify *Lmx1a*-expressing cells.

Coronal section of e18.5 *Lmx1a-Cre;ROSA-YFP* telencephalon stained with DAPI and indicated antibodies. Panels **b, d-f** show higher magnification of regions boxed in panel **a**. Panel **c** shows higher magnification of region boxed in panel **b**.

In the telencephalon of *Lmx1a-Cre* mice, Cre is specifically expressed in Lmx1a+ cells[1]. In *Lmx1a-Cre;ROSA-YFP* mice, cells that at any point during development expressed *Lmx1a* or those originating from *Lmx1a*-expressing progenitors were YFP+.

YFP expression was found in ChPe (**a** and arrows in **d-f**). Mesenchyme adjacent to ChPe was YFP-negative (**d-f**, arrowheads). YFP was expressed in fimbria (fim.) (**a**) and in few cells located deep in the hippocampus (**a**, open arrowhead). In addition, YFP+ cells occupied the hippocampal fissure (**a**, arrow) and outer surface of the neocortex (**a** and **b**, white arrowheads). These cells are likely Cajal-Retzius (CR) cells because they were located exactly where CR cells are found in embryonic telencephalon[2] and CR cells are known to originate from *Lmx1a*-expressing CH[1]. In the neocortex, these superficially located YFP+ cells coexpressed a CR cell marker Reelin (**c**, arrowheads), further confirming that they were CR cells. No YFP expression was found in the neocortex beyond CR cells or in the mesenchyme (mes.) (**b**), confirming that in the embryonic telencephalon *Lmx1a* is specifically expressed in the DMe and its derivatives.

Scale bars: 200 µm (**a**); 100 µm (**b**); 50 µm (**c-f**).

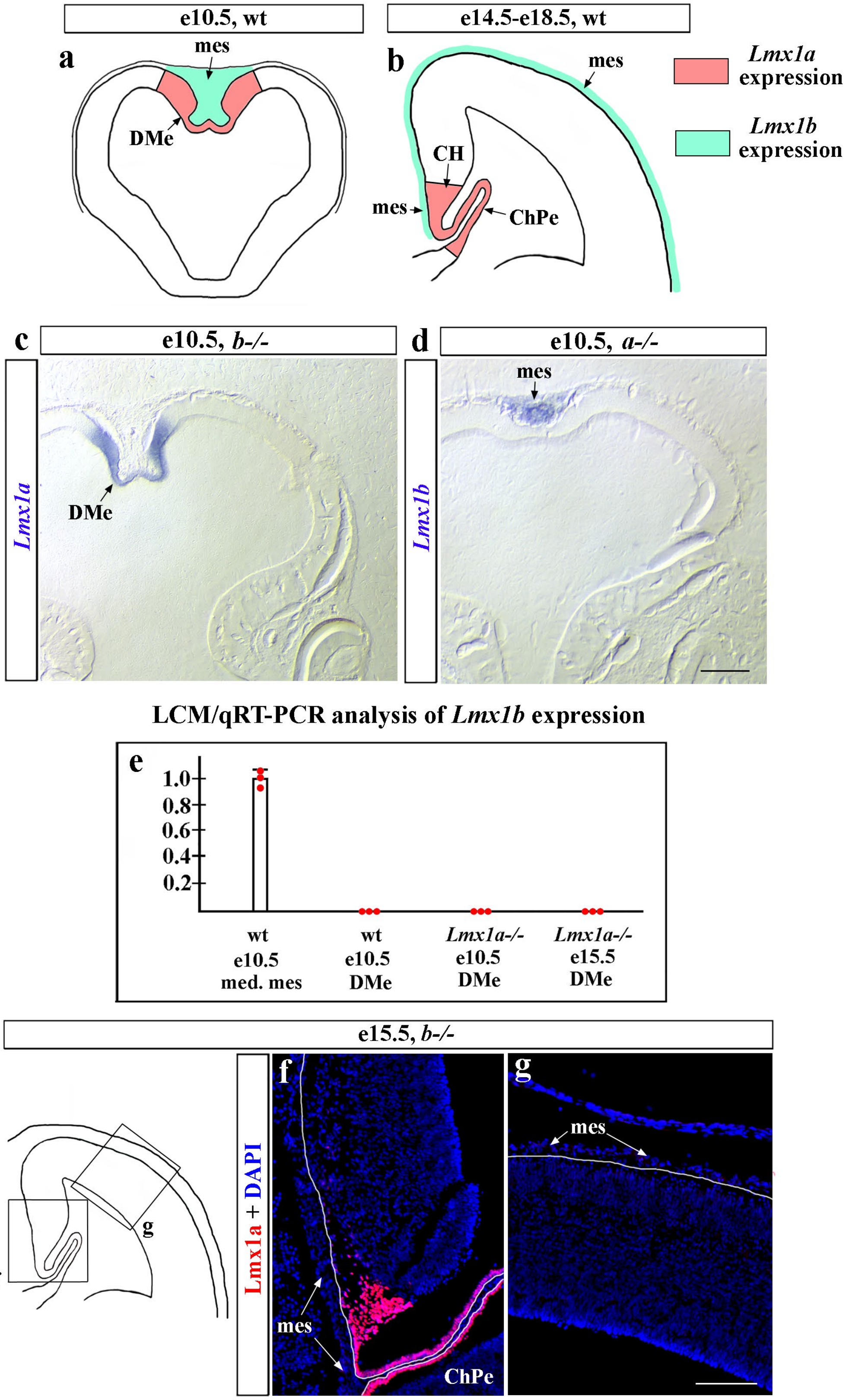

Supplementary Fig. 7. Loss of one *Lmx1* gene does not alter expression of the remaining *Lmx1* gene. (**a, b**) Diagrams summarizing expression of *Lmx1a* and *Lmx1b* in early and late wild-type embryonic telencephalon based on gene expression analysis described in **Supplementary Figures 5** and **6**.

(**c, d**) *In situ* hybridization showing that in e10.5 *Lmx1b-/-* embryos, *Lmx1a* is still properly expressed in DMe, and in e10.5 *Lmx1a-/-* embryos, *Lmx1b* is properly expressed in medial mesenchyme (mes.).
(**e**) Laser capture microdissection (LCM)/qRT-PCR analysis of *Lmx1b* expression. LCM was used to isolate telencephalic medial mesenchyme from e10.5 wild-type embryos (green domain in diagram in panel **a**), DMe from e10.5 wild-type and *Lmx1a-/-* embryos (red domain in diagram in panel **a**), and DMe from e15.5 *Lmx1a-/-* embryos (red domain in diagram in panel **b**). LCM-isolated tissue was analyzed by q-RT-PCR that revealed that loss of *Lmx1a* did not result in activation of *Lmx1b* expression in *Lmx1a*-expressing territory. Medial mesenchyme and DMe from e10.5 wild-type embryos were used as positive and negative controls, respectively.
(**f, g**) Immunohistochemistry revealed that in e15.5 *Lmx1b-/-* mutants, *Lmx1a* was properly expressed in DMe (ChPe and CH) but not in mesenchyme.
Source data for panel **e** is provided as Source Data File.
Scale bars: 200 μm (**c**, **d**); 100 μm (**f, g**)

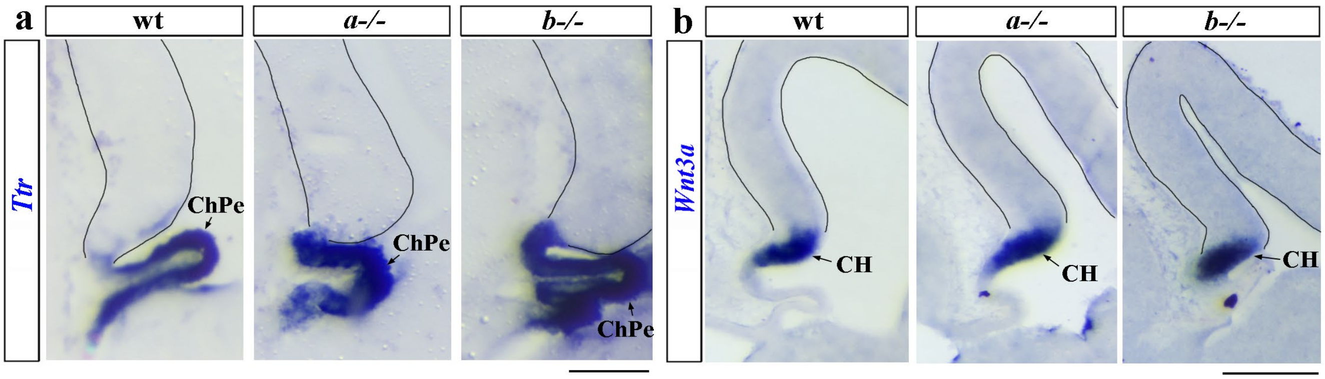

Supplementary Fig. 8. DMe patterning is normal in *Lmx1a* and *Lmx1b* single-gene mutants at e12.5. Telencephalic coronal sections labeled with *Ttr* (**a**) or *Wnt3a* (**b**) *in situ* probes. Similar to wild-type controls, in *Lmx1a* and *1b* single-gene mutants, *Ttr* was expressed in the ChPe and *Wnt3a* – in the CH, revealing normal patterning of the DMe.
Scale bars: 100 μm (**a**); 200 μm (**b**).

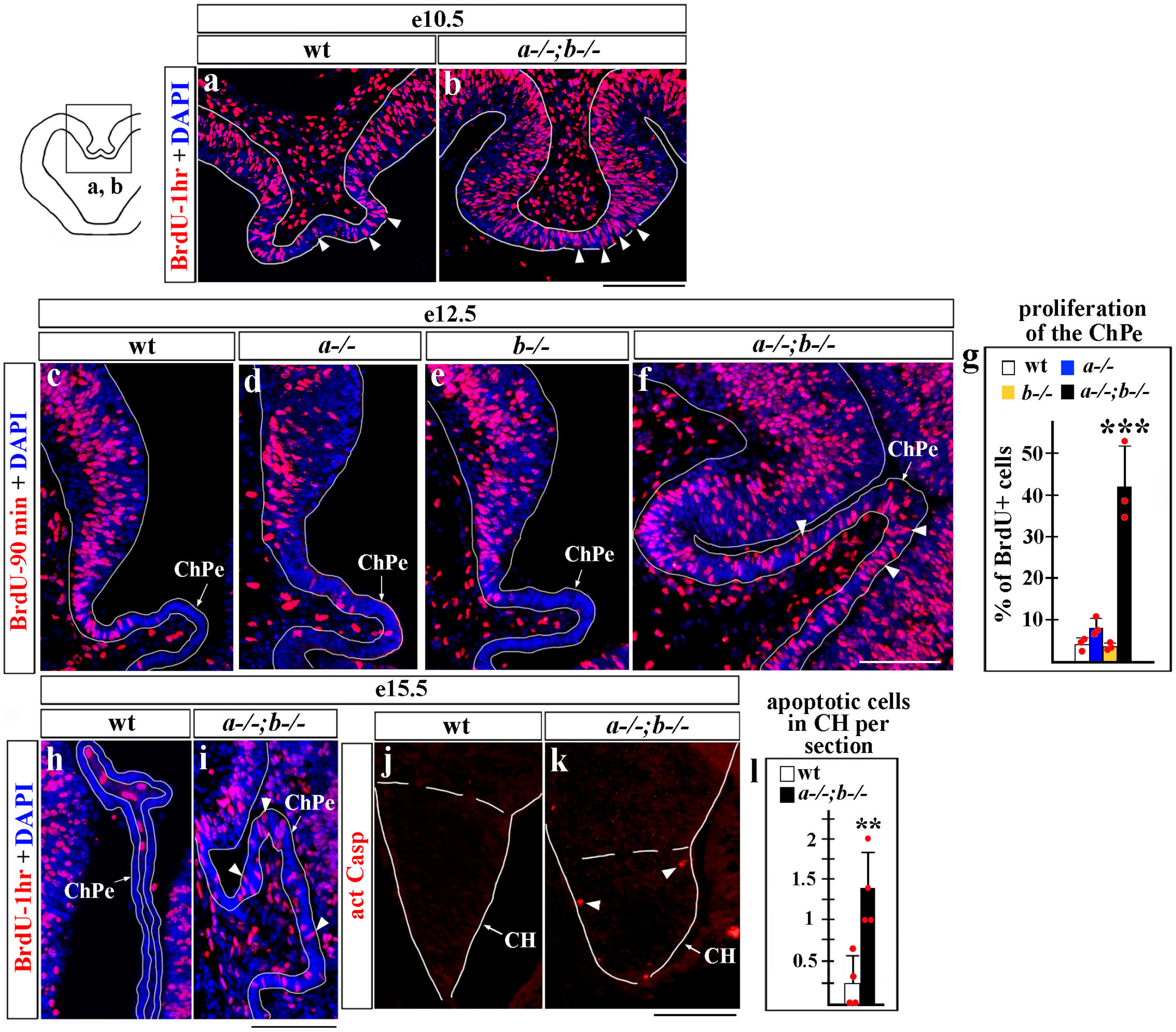

Supplementary Fig. 9. Proliferation of ChPe and apoptosis in late CH in *Lmx1a-/-;b-/-* embryos.
Telencephalic coronal sections labeled with DAPI and anti-BrdU antibody (**a-f, h, i**) or anti-activated Casp3 antibody (**j, k**). To reveal proliferating cells, mice were injected with BrdU 1 hour (**a, b, h, i**) or 90 min. (**c-f**) prior to collecting embryos.
(**a, b**) Compared to wild-type embryos, e10.5 *Lmx1a-/-;b-/-* DMe was abnormally thick and contained more BrdU+ (proliferating) cells (arrowheads).
(**c-f**) At e12.5, in contrast to wild-type and *Lmx1a* and *Lmx1b* single-gene mutants, in *Lmx1a/b* double mutants, numerous BrdU+ (proliferating) cells (**f**, arrowheads) were found in the ChPe. (**g**) Quantification of proliferating cells (the number of BrdU+ cells divided by the number of DAPI+ cells) in the ChPe (n=3 embryos per genotype, one-way ANOVA with Tukey's post hoc test, ***p<0.001 for *Lmx1a-/-;b-/-* versus wild-type, for *Lmx1a-/-;b-/-* versus *Lmx1a-/-*, and for *Lmx1a-/-;b-/-* versus *Lmx1b-/-* comparisons).
(**h, i**) Many cells were BrdU+ (proliferating) (arrowheads in **i**) in e15.5 *Lmx1a-/-;b-/-* but not wild-type ChPe.
(**j-l**) More apoptotic (Casp 3+) cells (arrowheads) were found in the *Lmx1a-/-;b-/-* CH at e15.5 compared to controls (n=4 embryos per genotype, two-tailed t-test, **p<0.01).
All data are mean ± sd. Source data for panels **g**, **l** are provided as Source Data File.
Scale bars: 100 µm.

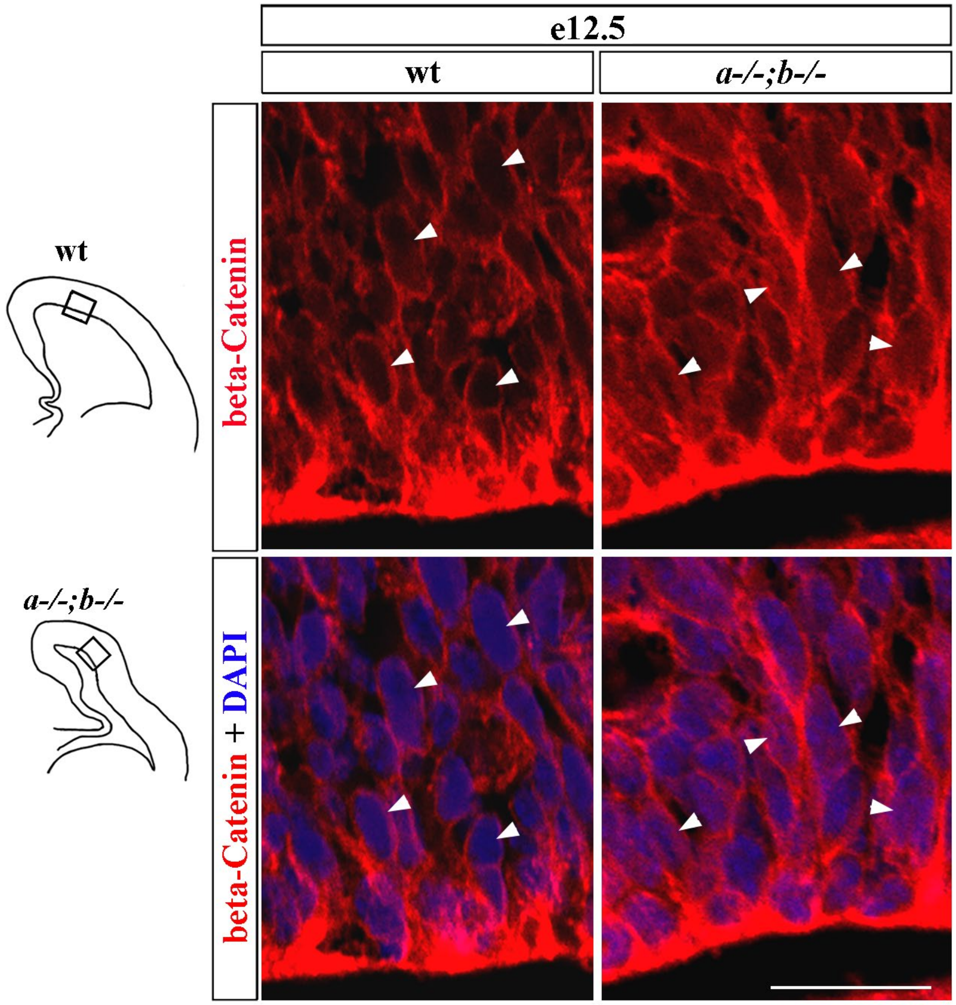

Supplementary Fig. 10. Elevated nuclear β-catenin in early *Lmx1a-/-;b-/-* neocortex.
Confocal immunofluorescence images of e12.5 coronal sections. Regions shown in panels correspond to areas boxed in adjacent diagrams. DAPI staining identifies nuclei. In wild-type neocortex, most cells did not reveal nuclear β-catenin staining (arrowheads), indicating low level of β-catenin signaling. In *Lmx1 a-/-;b-/-*, neocortex, nuclear β-catenin was visible in many cells (arrowheads), indicating a higher activity of the β-catenin signaling pathway.
Scale bar: 20 μm.

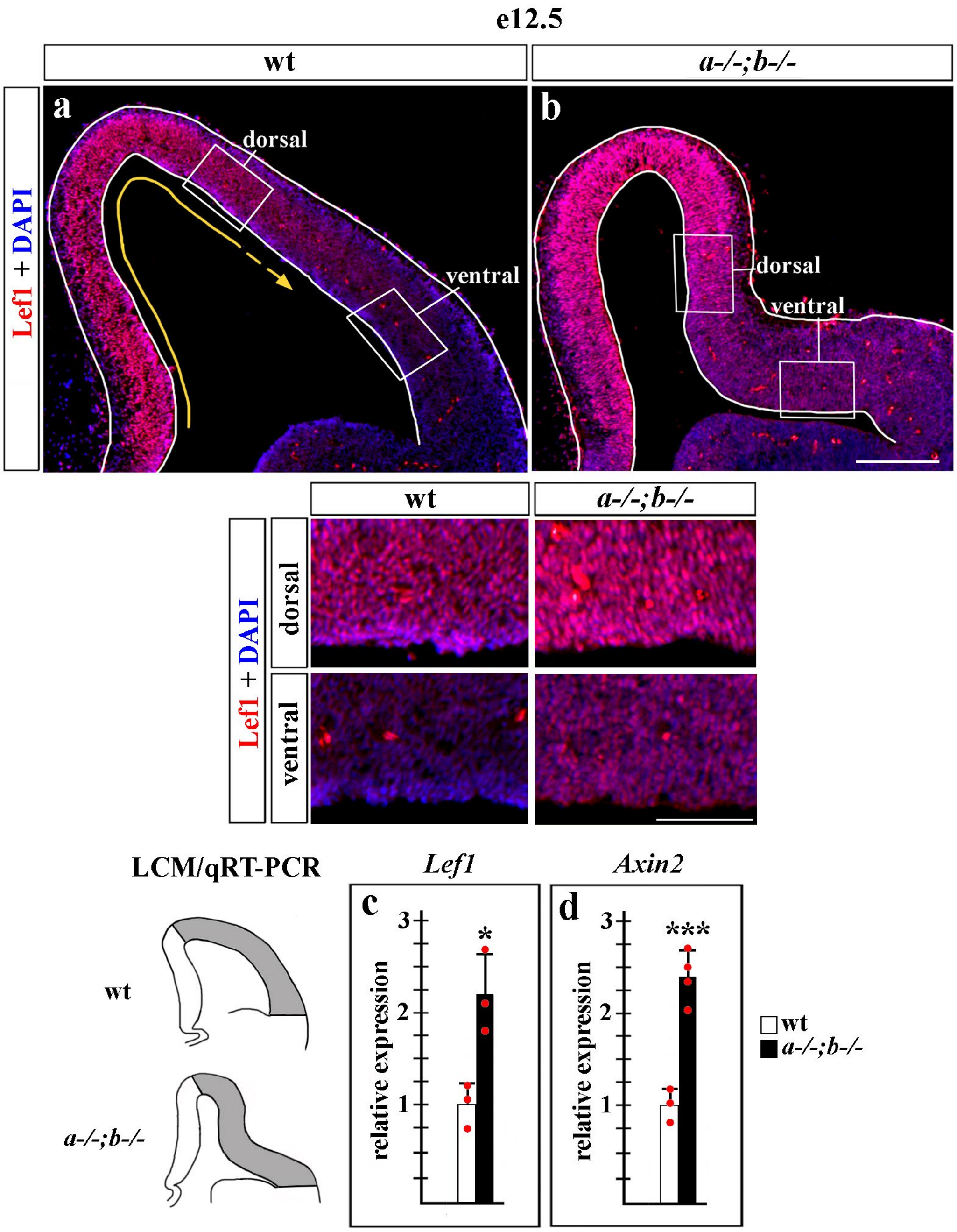

Supplementary Fig 11. Elevated *Lef1* and *Axin2* expression in early *Lmx1a-/-;b-/-* neocortex.
(**a, b**) Coronal section of e12.5 telencephalon stained with DAPI and an anti-Lef1 antibody. Insets show high magnification of dorsal and ventral regions of neocortical primordium boxed in corresponding low magnification panels. (**a**) Yellow arrow shows dorso-medial – high, lateral - low Lef1 (Wnt-β-catenin target gene) expression gradient in wild-type telencephalon. (**b**) A similar but brighter Lef1 expression gradient was present in *Lmx1a-/-;b-/-* telencephalon at e12.5.
(**c, d**) e12.5 cortical primordium (between the dorsal telencephalic bend and LGE, gray area in adjacent diagrams) was isolated by LCM and assayed by qRT-PCR. Expression of β-catenin downstream targets, *Lef1* and *Axin2,* was upregulated in *Lmx1a-/-;b-/-* cortex relative to wild-type cortex (n=3 embryos per genotype for *Lef1* analysis and n=3 wild-type embryos and n=4 *Lmx1a-/-;b-/-* embryos for *Axin2* analysis,

two-tailed t-test, *p<0.05, ***p<0.001), further confirming higher activity of the β-catenin pathway at this developmental stage. All data are mean ± sd. Source data for panels **c**, **d** are provided as Source Data File. Scale bar: 200 μm (**a, b**), 80 μm (**insets**).

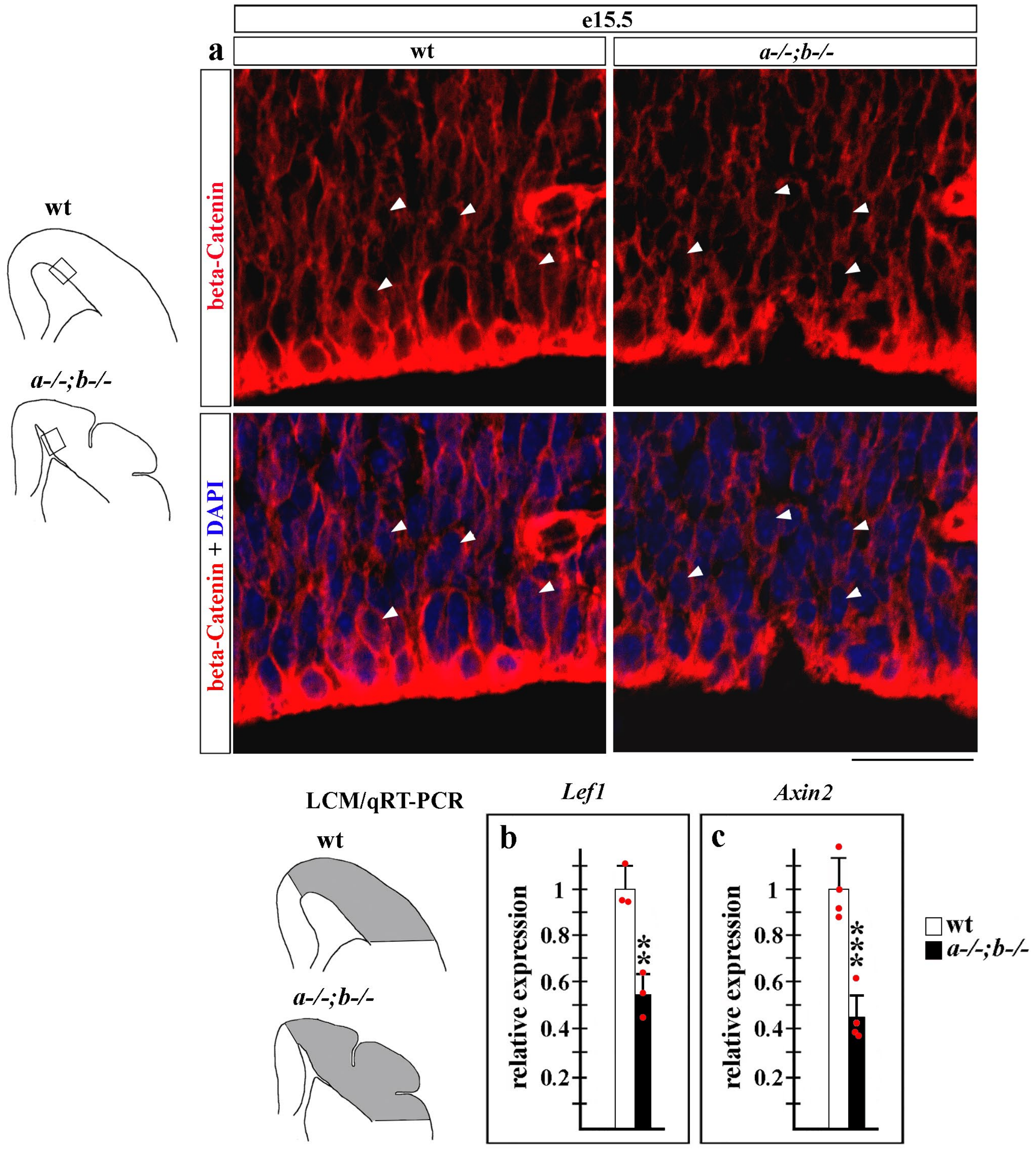

Supplementary Fig. 12. Reduced β-catenin signaling in e15.5 *Lmx1a-/-;b-/-* neocortex.
(**a**) Confocal immunofluorescence images of e15.5 coronal sections. Regions shown in panel **a** correspond to areas boxed in adjacent diagrams. DAPI staining identifies nuclei. Similar to wild-type embryos, in

Lmx1a-/-;b-/- neocortex, most cells did not reveal nuclear β-catenin staining (arrowheads), showing that in the double mutants, β-catenin signaling was not elevated at this developmental stage.
(**b**, **c**) e15.5 cortical primordium (between the dorsal telencephalic bend and LGE, gray area in adjacent diagrams) was isolated by LCM and assayed by qRT-PCR. Expression of both *Lef1* (**b**) and *Axin2* (**c**), was downregulated in *Lmx1a-/-;b-/-* cortex relative to wild-type cortex, arguing that β-catenin signaling was actually decreased in *Lmx1a-/-;b-/-* neocortex at e15.5 (n=3 embryos per genotype for *Lef1* analysis and n=4 embryos per genotype for *Axin2* analysis, two-tailed t-test, $**p<0.01$, $***p<0.001$).
All data are mean ± sd. Source data for panels **b**, **c** are provided as Source Data File.
Scale bar: 25 µm.

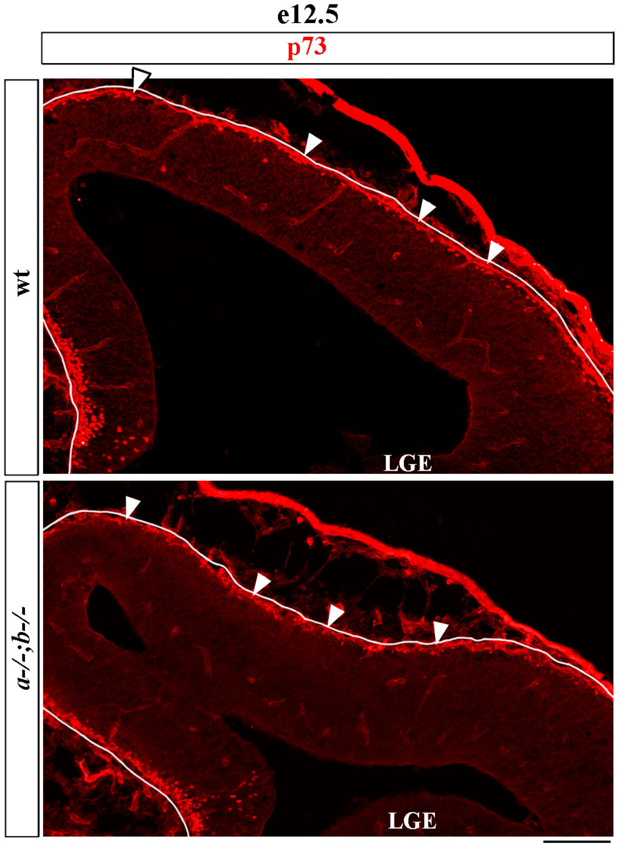

Supplementary Fig. 13. Cajal-Retzius cells appropriately cover e12.5 *Lmx1a-/-;b-/-* neocortex.
Coronal sections of wild-type and *Lmx1a-/-;b-/-* telencephalon immunostained against CR marker p73. Arrowheads point to p73+ CR cells that evenly cover the neocortical surface of both wild-type and *Lmx1 a-/-;b-/-* embryos. Scale bar: 100 µm.

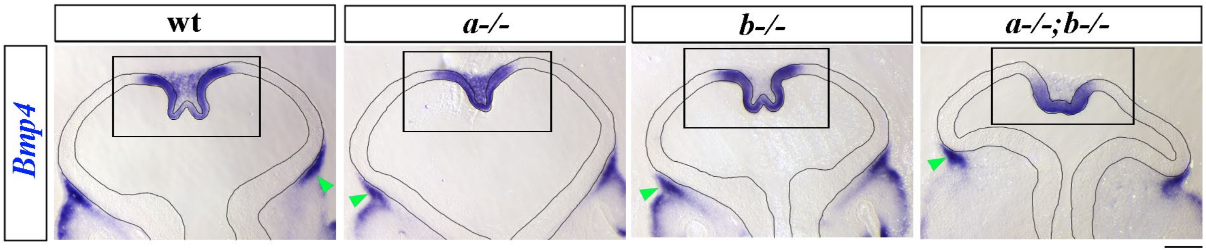

Supplementary Fig. 14. Low magnification images of e10.5 sections stained with *Bmp4 in situ* probe. Boxed regions correspond to high magnification images shown in **Fig. 5b-e**. Green arrowheads point to *Bmp4* expression in lateral mesenchyme, which was similar in all four genotypes.
Scale bar: 200 µm

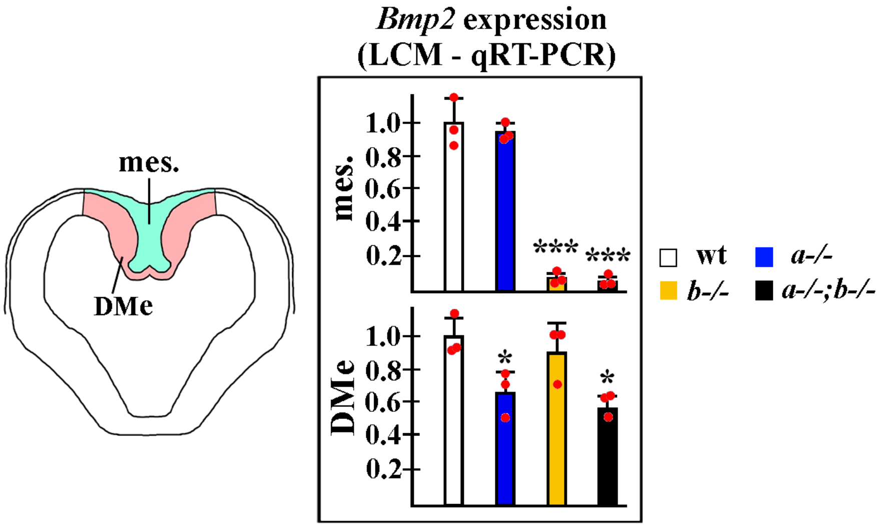

Supplementary Fig. 15. *Lmx1a* and *Lmx1b* control expression of *Bmp2* in their respective territories. Medial mesenchyme (green region in the schematic) and DMe (red region in the schematic) were isolated by LCM and assayed by qRT-PCR. Loss of *Lmx1b* reduced expression of *Bmp2* in medial mesenchyme (mes.), (n=3 embryos per genotype, one-way ANOVA with Tukey's post hoc test, ***p<0.001 for *Lmx1 b-/-* versus wild-type and *Lmx1a-/-;b-/-* versus wild-type comparisons). Loss of *Lmx1a* reduced expression of *Bmp2* in the DMe (n=3 embryos per genotype, one-way ANOVA with Tukey's post hoc test, *p<0.05 for *Lmx1a-/-* versus wild-type and *Lmx1a-/-;b-/-* versus wild-type comparisons).
All data are mean ± sd. Source data are provided as Source Data File.

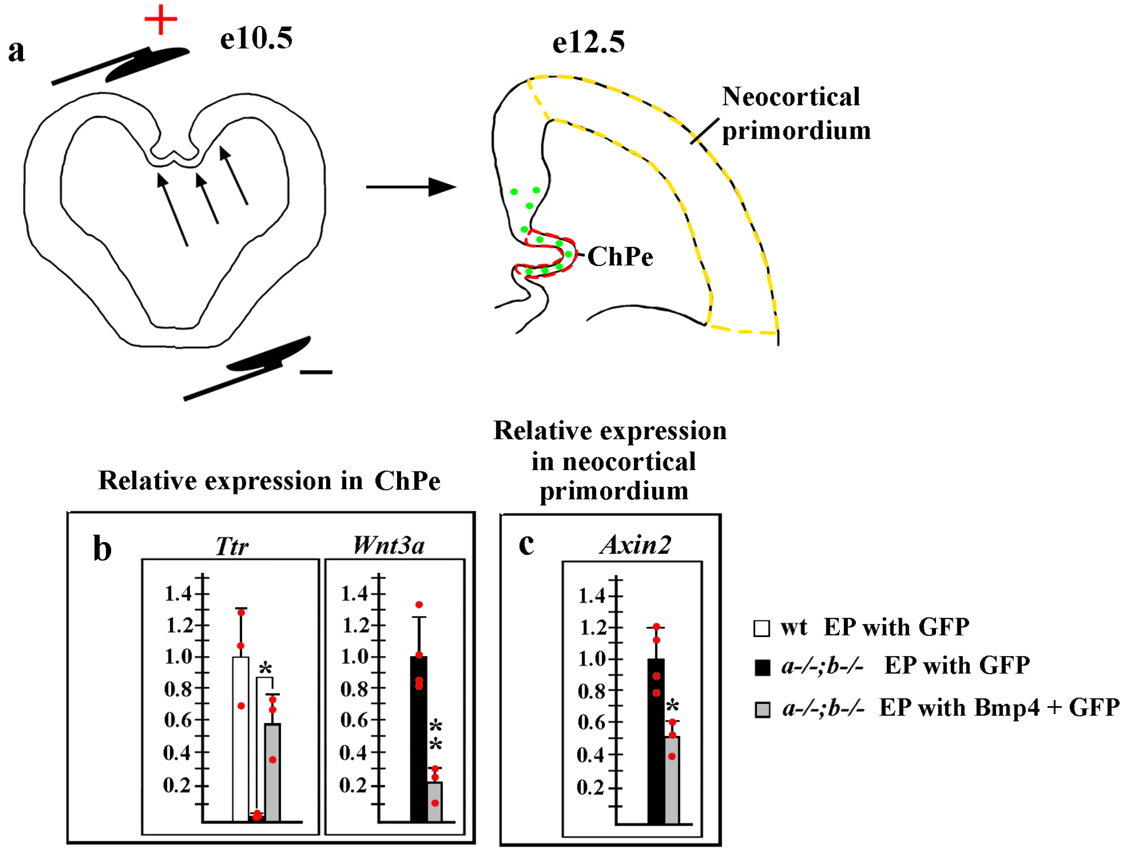

Supplementary Fig. 16. *Bmp4* induces *Ttr* and inhibits *Wnt* signaling in e12.5 *Lmx1a-/-;b-/-* mutants. (**a**) A diagram summarizing experimental approach. *Bmp4* and/or *GFP* expressing plasmids were electroporated into DMe *in utero* at e10.5 and embryos were analyzed at e12.5. To electroporate DMe, the positive electrode was placed against this region, as shown in the left schematic. Negatively charged DNA moves toward the positive electrode (arrows) entering DMe. Electroporated cells are shown as green circles in the right schematic. Embryos showing specific GFP expression in the DMe (similar to those in **Fig. 6e, f**) were used for analysis. Their ChPe (the area outlined by red dashed line in the right schematic) and neocortical primordium (the area between the dorsal telencephalic bend and LGE, outlined by yellow dashed line in the right schematic) were isolated by LCM and analyzed by qRT-PCR.
(**b, c**) Electroporation of *Bmp4* into DMe of *Lmx1a-/-;b-/-* mutants induced *Ttr* in the ChPe (n=3 embryos per condition, one-way ANOVA with Tukey's post hoc test, *p<0.05 for *Lmx1a-/-;b-/-* embryos electroporated with *GFP* versus *Lmx1a-/-;b-/-* embryos electroporated with *Bmp4* + *GFP*), downregulated *Wnt3a* in the ChPe (n=4 *Lmx1a-/-;b-/-* embryos electroporated with *GFP* versus n=3 *Lmx1a-/-;b-/-* embryos electroporated with *Bmp4* + *GFP*, two-tailed t-test, **p<0.01) and inhibited Wnt-β-catenin downstream target *Axin2* in distantly located neocortical primordium (n=4 *Lmx1a-/-;b-/-* embryos electroporated with *GFP* versus n=3 *Lmx1a-/-;b-/-* embryos electroporated with *Bmp4* + *GFP*, two-tailed t-test, *p<0.05).
All data are mean ± sd. Source data for panels **b**, **c** are provided as Source Data File.

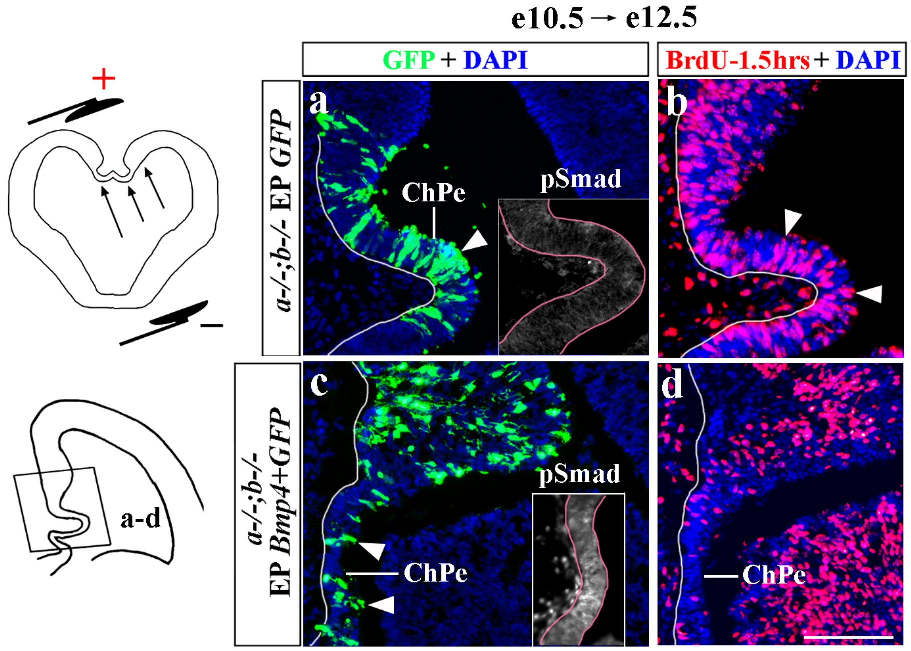

Supplementary Fig. 17. *Bmp4* reduces proliferation in the *Lmx1a-/-;b-/-* ChPe.
Lmx1a-/-;b-/- embryos were in *in utero* electroporated into the DMe at e10.5 (by placing the electrodes as shown in the upper schematic) and analyzed at e12.5. Panels **a-d** show dorsal midline region (boxed region in the lower schematic). Panels show sections immunostained against GFP, pSmad or BrdU.
Images shown in panels **a, a inset,** and **b**, and those in panels **c, c inset** and **d** are from adjacent sections, from the same experiment as shown in **Fig. 6**. Panels **a** and **c** showing expression of GFP in ChPe (arrowheads) have already been shown as **Fig. 6a, c**. They confirm that images **b** and **d** were indeed taken from embryos with electroporated DMe. Panels **a** and **c** were included here again for ease of comparison.
(**a, a inset, c, c inset**) Electroporation of *Bmp4* increased pSmad intensity in *Lmx1a-/-;b-/-* ChPe, demonstrating that even mosaic expression from this plasmid was sufficient to increase level of Bmp signaling.
(**b, d**) After a 1.5 hr. BrdU pulse, BrdU+ (proliferating) cells were much more numerous in *Lmx1a-/-;b-/-* ChPe electroporated with GFP (**b**, arrowheads) compared to *Lmx1a-/-;b-/-* ChPe electroporated with *Bmp4* (**d**). Scale bar: 100µm

Supplementary Table 1. Sequences of primers used in qRT-PCR

Primers	References
Lmx1b F: 5'-CAGCAGCGAAGAGCTTTCAA-3'<br>Lmx1b R: 5'-GTCTCTCGGACCTTCCGACA-3'	(3)
Bmp4 F: 5'-GAGGAGTTTCCATCACGAAGA-3'<br>Bmp4 R: 5'-GCTCTGCCGAGGAGATCA-3'	(4, 5)
Bmp2 F: 5'-AGATCTGTACCGCAGGCACT-3'<br>Bmp2 R: 5'-GTTCCTCCACGGCTTCTTC-3'	(6)
Lef1 F: 5'-GGCCAGAGAACACCCTGAT-3'<br>Lef1 R: 5'-GCTGTTCATATTGGGCATCAT-3'	This study
Axin2 F: 5'-CAAGCCTGGCTCCAGAAG-3'<br>Axin2 R: 5'-AAAAGTAGGTGACAACCAGCTCA-3'	This study
Ttr F: 5'-AAAAGACCTCTGAGGGATCCT-3'<br>Ttr R: 5'- GGTACAAATGGGATGCTACTGC-3'	(7)
Wnt3a F: 5'-CTCCTCTCGGATACCTCTTAGTG-3'<br>Wnt3a R: 5'-GCATGATCTCCACGTAGTTCCTG-3'	(8)
Gapdh F: 5'-CGACTTCAACAGCAACTCCCACTCTTCC-3'<br>Gapdh R: 5'-TGGGTGGTCCAGGGTTTCTTACTCCTT-3'	(9)

Supplementary References

1. Chizhikov, V. V. et al. Lmx1a regulates fates and location of cells originating from the cerebellar rhombic lip and telencephalic cortical hem. *Proc. Natl. Acad. Sci. USA.* **107**, 10725-30 (2010).
2. Hodge, R. D. et al. Tbr2 expression in Cajal-Retzius cells and intermediate neuronal progenitors is required for morphogenesis of the dentate gyrus. *J. Neurosci.* **33**, 4165-80 (2013).
3. Suleiman, H. et al. The podocyte-specific inactivation of Lmx1b, Ldb1 and E2a yields new insight into a transcriptional network in podocytes. *Dev. Biol.* **304**, 701-12 (2007).
4. Jung, Y. K. et al. Role of interleukin-10 in endochondral bone formation in mice: anabolic effect via the bone morphogenetic protein/Smad pathway. *Arthritis Rheum.* **65**, 3153-64 (2013).
5. Ma, X. et al. ISL1 regulates peroxisome proliferator-activated receptor γ activation and early adipogenesis via bone morphogenetic protein 4-dependent and -independent mechanisms. *Mol. Cell. Biol.* **34**, 3607-17 (2014).
6. Liao, K. H. et al. Endothelial angiogenesis is directed by RUNX1T1-regulated VEGFA, BMP4 and TGF-β2 expression. *PLoS One* **12**, e0179758 (2017).
7. Wang, X. et al. The systemic amyloid precursor transthyretin (TTR) behaves as a neuronal stress protein regulated by HSF1 in SH-SY5Y human neuroblastoma cells and APP23 Alzheimer's disease model mice. *J. Neurosci.* **34**, 7253-65 (2014).
8. Watanabe, M. et al. BMP4 acts as a dorsal telencephalic morphogen in a mouse embryonic stem cell culture system. *Biol. Open* **5**, 1834-1843 (2016).
9. Liu, G. et al. miR-147, a microRNA that is induced upon Toll-like receptor stimulation, regulates murine macrophage inflammatory responses. *Proc. Natl. Acad. Sci. USA* **106**, 15819-24 (2009).
