# Supplementary material for: Early dorsomedial tissue interactions regulate gyrification of distal neocortex
Source: Nat Commun. 2019 Nov 15;10:5192. doi: 10.1038/s41467-019-12913-z (PMC6858446; doi:10.1038/s41467-019-12913-z)
Supplement: Supplementary file 2 — Reporting Summary [file 41467_2019_12913_MOESM2_ESM.pdf]

nature research

Corresponding author(s): Victor V. Chizhikov and Kathleen J. Millen

Last updated by author(s): Sep 10, 2019

Reporting Summary

Nature Research wishes to improve the reproducibility of the work that we publish. This form provides structure for consistency and transparency in reporting. For further information on Nature Research policies, see Authors & Referees and the Editorial Policy Checklist .

Statistics

For all statistical analyses, confirm that the following items are present in the figure legend, table legend, main text, or Methods section.

n/a	Confirmed	
☐	☒	The exact sample size (*n*) for each experimental group/condition, given as a discrete number and unit of measurement
☐	☒	A statement on whether measurements were taken from distinct samples or whether the same sample was measured repeatedly
☐	☒	The statistical test(s) used AND whether they are one- or two-sided<br>*Only common tests should be described solely by name; describe more complex techniques in the Methods section.*
☒	☐	A description of all covariates tested
☐	☒	A description of any assumptions or corrections, such as tests of normality and adjustment for multiple comparisons
☐	☒	A full description of the statistical parameters including central tendency (e.g. means) or other basic estimates (e.g. regression coefficient) AND variation (e.g. standard deviation) or associated estimates of uncertainty (e.g. confidence intervals)
☐	☒	For null hypothesis testing, the test statistic (e.g. *F*, *t*, *r*) with confidence intervals, effect sizes, degrees of freedom and *P* value noted<br>*Give P values as exact values whenever suitable.*
☒	☐	For Bayesian analysis, information on the choice of priors and Markov chain Monte Carlo settings
☒	☐	For hierarchical and complex designs, identification of the appropriate level for tests and full reporting of outcomes
☒	☐	Estimates of effect sizes (e.g. Cohen's *d*, Pearson's *r*), indicating how they were calculated

Our web collection on statistics for biologists contains articles on many of the points above.

Software and code

Policy information about availability of computer code

Data collection	images were taken using AxioVision Rel 4.9 software (Zeiss) or LAS V3.8 and V4.5 software (Leica).
Data analysis	Image J (NIH), IBM SPSS Statistics (version 25), Microsoft Excel 2013, Photoshop CC 2018 (Adobe).

For manuscripts utilizing custom algorithms or software that are central to the research but not yet described in published literature, software must be made available to editors/reviewers. We strongly encourage code deposition in a community repository (e.g. GitHub). See the Nature Research guidelines for submitting code & software for further information.

Data

Policy information about availability of data

All manuscripts must include a data availability statement. This statement should provide the following information, where applicable:

- Accession codes, unique identifiers, or web links for publicly available datasets
- A list of figures that have associated raw data
- A description of any restrictions on data availability

The data that support the findings of this study are available from the corresponding authors upon reasonable request. Source data for Figs. 1k, n; 2f, i, l, o, r, u; 4f-h; 5f, k and Suppl. Figs. 1f-h; 3k-o, r; 4d; 7e; 9g, l; 11c, d; 12b, c; 15 and 16b, c are provided as a Source data file.

Field-specific reporting

Please select the one below that is the best fit for your research. If you are not sure, read the appropriate sections before making your selection.

☒ Life sciences ☐ Behavioural & social sciences ☐ Ecological, evolutionary & environmental sciences

Life sciences study design

All studies must disclose on these points even when the disclosure is negative.

Sample size	Sample-size calculation was not performed. Sample sizes were chosen based on published studies that used the same techniques and are reported in figure legends.
Data exclusions	In utero electroporated embryos that did not show GFP expression or showed GFP expression in non-desired brain regions were excluded from the study.
Replication	All experimental replications were successful.
Randomization	Randomization was not relevant to this study. Embryos were allocated into age matched groups based on genotypes. When possible, littermates were used.
Blinding	Investigators were not blinded.

Reporting for specific materials, systems and methods

We require information from authors about some types of materials, experimental systems and methods used in many studies. Here, indicate whether each material, system or method listed is relevant to your study. If you are not sure if a list item applies to your research, read the appropriate section before selecting a response.

Materials & experimental systems

n/a	Involved in the study
☐	☒ Antibodies
☒	☐ Eukaryotic cell lines
☒	☐ Palaeontology
☐	☒ Animals and other organisms
☒	☐ Human research participants
☒	☐ Clinical data

Methods

n/a	Involved in the study
☒	☐ ChIP-seq
☒	☐ Flow cytometry
☒	☐ MRI-based neuroimaging

Antibodies

Antibodies used	The following primary antibodies were used: anti-Lmx1a (goat, Santa-Cruz, catalog #sc-54273, 1:500, RRID:AB_2136830), anti-Cux1 (rabbit, Santa-Cruz, catalog #sc-13024, 1:200, RRID:AB_2261231), anti-Ctip2 (rat, Abcam, catalog #ab18465, 1:500, RRID:AB_2064130), anti-GFP antibody that recognizes YFP (chicken, catalog #ab13970 Abcam, 1:500, RRID:AB_300798), anti-Reelin (mouse, Chemicon, catalog #Mab5364, 1:500, RRID:AB_2179313), anti-BrdU (rat, Abcam, catalog #ab6326, 1:50, RRID:AB_305426), anti-BrdU (mouse, Rockland, catalog #600-401-c29, 1:300, RRID:AB_10893609), anti-Ki67 (mouse, BD Pharmingem, catalog #556003, 1:250, RRID:AB_396287), anti-Pax6 (rabbit, Covance, catalog #Prb-278p-100, 1:300, RRID:AB_291612), anti-Tbr2 (rat, eBioscience, catalog #14-487582, 1:200, RRID:AB_11042577), anti-β-catenin (mouse, BD Biosciences, catalog #610153, 1:200, RRID:AB_397554), anti-Lef1 (rabbit, Cell Signaling Technology, catalog #2230S, 1:300) anti-pSmad (rabbit, Chemicon, catalog #AB3848, 1:50, RRID:AB_177439), anti-Ttr (rabbit, Dako, catalog #A0002, 1:200, RRID:AB_2335696), anti-activated Caspase 3 (rabbit, Promega, catalog #G7481, 1:250, RRID:AB_430875), and anti-p73 (mouse, Fisher, catalog #MA5-14117, 1:100, RRID:AB_10987160) with species-specific secondary antibodies conjugated with Alexa 350, 488, 568 or 594 fluorophores (Life Technologies).
Validation	Antibodies were validated based on known expression from previously published studies and/or by the suppliers.

Animals and other organisms

Policy information about studies involving animals; ARRIVE guidelines recommended for reporting animal research

Laboratory animals	Lmx1a-null (drJ, The Jackson Laboratory strain #000636), Lmx1b-null (Lmx1b-/-), Lmx1bLacZ, Lmx1a-Cre and ROSA26-YFP (The Jackson Laboratory strain #006148) mice were used in this study. Mice were maintained on a mixed genetic background comprising C57Bl6, 129, FVB and CD1. Embryos (e8.5-e18.5) of both sexes were used for experiments.
Wild animals	N/A
Field-collected samples	N/A
Ethics oversight	All animal experiments were approved by the Institutional Animal Care and Use Committees of the University of Tennessee Health Science Center (UTHSC), Seattle Children's Research Institute or the University of Chicago.

Note that full information on the approval of the study protocol must also be provided in the manuscript.
